# IKKβ-mediated inflammatory myeloid cell activation exacerbates experimental autoimmune encephalomyelitis by potentiating Th1/Th17 cell activation and compromising blood brain barrier

**DOI:** 10.1186/s13024-016-0116-1

**Published:** 2016-07-22

**Authors:** Min Jung Lee, So Jin Bing, Jonghee Choi, Minhee Jang, Gihyun Lee, Hyunkyoung Lee, Byung Soo Chang, Youngheun Jee, Sung Joong Lee, Ik-Hyun Cho

**Affiliations:** Department of Convergence Medical Science, College of Korean Medicine, Kyung Hee University, Seoul, 02447 Republic of Korea; Brain Korea 21 Plus Program, College of Korean Medicine, Kyung Hee University, Seoul, 02447 Republic of Korea; Department of Veterinary Medicine, Jeju National University, Jeju, 63243 Republic of Korea; Department of Physiology, College of Korean Medicine, Kyung Hee University, Seoul, 02447 Republic of Korea; Department of Neuroscience and Dental Research Institute, School of Dentistry, Seoul National University, Seoul, 08826 Republic of Korea; Department of Cosmetology, Hanseo University, Seosan, 31962 Republic of Korea; Institute of Korean Medicine, College of Korean Medicine, Kyung Hee University, Seoul, 02447 Republic of Korea

**Keywords:** Myeloid cell, IKKβ conditional deletion, Experimental autoimmune encephalomyelitis, T cell, Blood brain barrier

## Abstract

**Background:**

The inflammatory myeloid cell activation is one of the hallmarks of experimental autoimmune encephalomyelitis (EAE), yet the in vivo role of the inflammatory myeloid cell activation in EAE has not been clearly resolved. It is well-known that IKK/NF-κB is a key signaling pathway that regulates inflammatory myeloid activation.

**Methods:**

We investigated the in vivo role of inflammatory myeloid cell activation in myelin oligodendrocyte glycoprotein (MOG) peptides-induced EAE using myeloid cell type-specific *ikkβ* gene conditional knockout-mice (*LysM-Cre/Ikkβ*^*F/F*^).

**Results:**

In our study, *LysM-Cre/Ikkβ*^*F/F*^ mice had alleviated clinical signs of EAE corresponding to the decreased spinal demyelination, microglial activation, and immune cell infiltration in the spinal cord, compared to the wild-type mice (WT, *Ikkβ*^*F/F*^). Myeloid *ikkβ* gene deletion significantly reduced the percentage of CD4^+^/IFN-γ^+^ (Th1) and CD4^+^/IL-17^+^ (Th17) cells but increased the percentages of CD4^+^/CD25^+^/Foxp3^+^ (Treg) cells in the spinal cord and lymph nodes, corresponding to the altered mRNA expression of IFN-γ, IL-17, IL-23, and Foxp3 in the spinal cords of *LysM-Cre/Ikkβ*^*F/F*^ EAE mice. Also, the beneficial effect of myeloid IKKβ deletion in EAE corresponded to the decreased permeability of the blood brain barrier (BBB).

**Conclusions:**

Our findings strongly suggest that IKK/NF-kB-induced myeloid cell activation exacerbates EAE by activating Th1 and Th17 responses and compromising the BBB. The development of NF-κB inhibitory agents with high efficacy through specific targeting of IKKβ in myeloid cells might be of therapeutic potential in MS and other autoimmune disorders.

**Electronic supplementary material:**

The online version of this article (doi:10.1186/s13024-016-0116-1) contains supplementary material, which is available to authorized users.

## Background

Multiple sclerosis (MS) is an autoimmune, demyelinating disease resulting from chronic inflammation in the central nervous system (CNS). The pathological features of MS are characterized by breakdown of the blood-brain barrier (BBB), infiltration/recruitment of peripheral immune cells consisting of lymphocytes and macrophages, demyelination, axonal- and oligodendrocyte loss, and glial scar formation [1, 2, 14, 31, 42]. These pathological features of MS can be recapitulated in the animal model for experimental autoimmune encephalomyelitis (EAE). A series of studies have demonstrated that myeloid cells in the spinal cord, namely microglia and blood-derived macrophages, play pivotal roles in the pathogenesis of MS and EAE. Studies thus far indicate that the activation of these myeloid cells contribute to MS and EAE lesion formation, as they phagocytose myelin, leading to extensive myelin damage and oligodendrocyte dysfunction [20]. In addition, activated microglia and brain-infiltrating macrophages in the demyelinated lesions produce inflammatory mediators including proinflammatory cytokines/chemokines, nitric oxide, and reactive oxygen species, which exert detrimental effects on MS and EAE by directly affecting oligodendrocyte cell death and/or recruiting autoreactive lymphocytes [20, 48, 57]. However, there are also documents indicating that these activated microglia/macrophages can be beneficial in the recovery process after EAE [31, 47, 48]. For instance, alternatively activated microglia and macrophages facilitate recovery from EAE by driving oligodendrocyte differentiation during remyelination [44]. In addition, efficient clearance of myelin debris and apoptotic cells in the lesion site and secretion of neuroprotective mediators from activated microglia/macrophages may promote regeneration of the impaired axons [47]. Thus, the in vivo role of macrophage/microglia activation in EAE is still controversial. Furthermore, recent studies revealed that microglia/macrophages display remarkable plasticity in their activation phenotypes. They can be activated as the “classically activated” M1 phenotype that produces and releases proinflammatory mediators [40] or the “alternatively activated” M2 phenotype that promotes angiogenesis and matrix remodeling [55] depending on their microenvironment. Therefore, the specific function of microglia/macrophage activation in the pathogenesis of EAE should be characterized according to the activation phenotype of these cells.

The nuclear factor (NF)-κB signaling pathway plays a central role in the expression of a wide variety of genes controlling immune and inflammatory responses. Upon stimulation, the canonical NF-κB pathway is activated by the IκB kinase (IKK) complex, which is composed of two catalytic subunits, IKKα and IKKβ [28, 41]. In microglia and macrophages, IKK/NF-κB-dependent signaling pathways are critical for M1 phenotype-related proinflammatory gene expression such as inducible nitric oxide synthase (iNOS) and tumor necrosis factor (TNF)-α [28, 41]. Due to its pivotal role in inflammatory myeloid cell activation, we reasoned that by selectively inhibiting the IKK/NF-κB signaling pathway in macrophages/microglia, we would be able to address the in vivo function of inflammatory myeloid cell activation in EAE. For the targeted analysis of NF-κB signaling, we used IKKβ-conditional knockout mice, in which the *ikkβ* gene is specifically deleted in myeloid cells, including the majority of microglia and macrophage populations [9, 18], and investigated the in vivo role of the IKK/NF-κB-dependent inflammatory myeloid cell activation during the complex process of demyelination through the development and progression of EAE. Our results showed that IKK/NF-κB-dependent proinflammatory myeloid cell activation exacerbates autoimmmune demyelination, Th17 cell infiltration, and BBB compromise during EAE. These data suggest that pharmacological targeting of the IKK/NF-κB signaling pathway, specifically in myeloid cells, might have therapeutic benefits in autoimmune demyelinating disorders including MS.

## Methods

### Animals, genotyping, and ethic statements

Myeloid cell type-specific IKK-β-deficient (*LysM-Cre/Ikkβ*^*F/F*^) mice were generated by crossing floxed-*ikkβ* (*Ikkβ*^*F/F*^) mice and LysM-Cre knock-in mice expressing Cre under the control of the endogenous lysozyme M promoter, as described previously [9, 10, 37]. The genetic background of both mice was C57BL/6. PCR genotyping was performed using the primers 5′-TGA CCC GGG AAT GAA TAG GA-3′ and 5′-GTC TTC AAC CTC CCA AGC CTT-3′, which amplify both the *Ikkβ*^*+*^ (220 bp) and *Ikkβ*^*F*^ (310 bp) alleles. *LysM-Cre* mice were genotyped by PCR using the primer pair NLS-Cre (5′-CCC AAG AAG AAG AGG AAG GTG TCC-3′) and Cre8 (5′-CCC AGA AAT GCC AGA TTA CG-3′) as previously described [9]. Adult (10–11 weeks after birth) female *LysM-Cre/Ikkβ*^*F/F*^ and wild-type (WT, *Ikkβ*^*F/F*^) mice were used.

Mice were housed at 23 ± 3 °C with a 12 h light–dark cycle (light from 08:00 to 20:00) and food and water were provided *ad libitum*. All experimental procedures were reviewed and approved by the Institutional Animal Care and Use Committee (IACUC) of Kyung Hee University and Seoul National University. Proper randomization of laboratory animals and handling of data were performed in a blinded manner in accordance with recent recommendations from a NIH Workshop on preclinical models of neurological diseases [30].

### Microglia isolation by CD11b microbeads

Microglial cells were isolated from the lumbar spinal cord of mice using CD11b microbeads (Miltenyil Biotec, Germany). Briefly, spinal cords were dissected after perfusion with PBS and single-cell suspensions were prepared by using the neural tissue dissociation Kit (Miltenyil Biotec) for 30 min at 37 °C. Pure single-cells were acquired by passing the cells through a 40 μm strainer. Myelins were removed by using myelin removal beads (Miltenyil Biotec) for 15 min at 4 °C. Single cells were stained with anti-CD11b antibody (Miltenyi Biotec) for 30 min at 4 °C. The CD11b^+^ labeled single cells were separated by a magnetic field through MS columns (Miltenyi Biotec). We confirmed that these cells were approximately 95 % pure, as assessed by CD11b^+^ and CD45^+^ cytometry analysis. Isolated microglia cells were used in real-time reverse transcription (RT)-polymerase chain reaction (PCR) analysis to investigate the level of *IKKβ* deletion in spinal microglia, as previously described [26], using the primer summarized in Additional file 1.

### Isolation of peritoneal macrophages and lipopolysaccharide-stimulation

Two ml of 2 % thioglycollate (BD Bioscience) was intraperitoneally administered to adult mice (*n* = 5 per group), to collect peritoneal macrophages. After 3–4 days, the peritoneal macrophages were harvested and isolated. The macrophages were distributed in Eagle’s minimal essential medium (EMEM) supplemented with 10 % heat-inactivated fetal bovine serum (FBS), seeded onto 24 plates (2.5 × 10^5^ cells/well), and incubated for 3 h at 37 °C in an atmosphere of 5 % CO_2_. After 4 h, non-adherent cells were removed by washing with Hanks balanced salt solution (HBSS) and the adherent cells (macrophages) were equilibrated with DMEM containing 10 % FBS before incubation with lipopolysaccharide (0111:B4; 100 ng/ml; Sigma-Aldrich) for 6 h. The macrophages were collected to analyze the mRNA levels of cytokines, including TNF-α, IL-1β, IL-6, and iNOS by real time RT-PCR using the primers summarized in Additional file 1.

### M1/M2 polarization of peritoneal macrophages

To analyze M1/M2 polarization of macrophage cells, macrophages equilibrated with DMEM were treated with lipopolysaccharide (0127:B8; 50 ng/ml; Sigma-Aldrich) for 4 h or IL-4 (0.1 ng/ml; R&D system) for 24 h. These macrophages were incubated with FITC anti-mouse F4/80 (F480, eBioscience), PE anti-mouse CD80 (B7-1; eBioscience), APC anti-mouse CD86 (B7-2, eBioscience), and APC anti-mouse CD206 (MMR, Biolegend) (Additional file 2) for 30 min at 4 °C. After washing twice with 2 % FBS in PBS, macrophages were used for flow cytometry analysis. Data were collected on a FACS Calibur flow cytometer (BD Biosciences) and analyzed using Cell Quest Pro software (BD Biosciences). Alteration in protein expression of M1 (iNOS) and M2 marker (arginine-1) was analyzed using protocol as described in 2.11. Western blot analysis.

### Preparation of spinal cord for real time RT-PCR and Western blot analyses

At the peak day of neurological impairment (15–18 days post-immunization), the mice used for real-time RT-PCR and Western blot analysis (*n* = 5 per group) were anesthetized. Each lumbar spinal cord was removed and deep-frozen.

### Real-time RT-PCR

Real-time RT-PCR was performed using SYBR Green PCR Master Mix (ABI, Warrington, UK) as described previously [9, 25, 34]. Reactions were performed in duplicate in a total volume of 10 μl containing 10 pM primer, 4 μl cDNA, and 5 μl SYBR Green PCR Master Mix. The mRNA levels of each target gene were normalized to that of GAPDH mRNA. Fold-induction was calculated using the 2^−∆∆CT^ method, as previously described [39]. All real-time RT-PCR experiments were performed at least thrice and the mean ± SEM values are presented unless otherwise noted. The PCR primer sequences used in this study are listed in Additional file 1.

### Histopathological analysis of spinal cord

Histopathological analysis of the spinal cord was performed as described previously [9, 23, 33, 34]. Detailed methods can be found in the Additional file 3.

### EAE induction and clinical evaluation

EAE induction and clinical evaluation was performed as previously described [32–34]. Detailed methods can be found in the Additional file 3.

### Quantification of demyelination and cell recruitment/infiltration

Methods for quantification of demyelination and cell recruitment/infiltration are presented in Additional file 3.

### Immunohistochemical analysis

Immunohistochemical analysis was performed as previously described [9, 25, 33, 34], using antibodies in Additional file 2. Detailed methods can be found in the Additional file 3.

### Western blot analysis

Western blot analysis was performed as previously described [25, 26, 33, 34], using antibodies in Additional file 2. Detailed methods can be found in the Additional file 3.

### Measurement of weights of spleen and lymph nodes

At the peak day of neurological impairment, five mice in each group were anesthetized. Their spleen and lymph nodes were carefully removed without fat, connective tissue, or fluid. They were weighted using a microbalance (OHAUS, Parsippany, U.S.A.).

### Flow cytometry

For flow cytometry analysis, 5 mice in each group were anesthetized. The lumbar spinal cord and lymph nodes were carefully dissected and dissociated at the time of the peak clinical score as previously described [23, 33, 34]. A single-cell suspension was prepared and fixed with 2 % paraformaldehyde for surface cell analysis. Cells were then washed with 2 % FBS in PBS, incubated with mouse anti-rat CD32 (BD Biosciences, San Jose, CA, USA) for 10 min to block the Fc receptor and washed twice with 2 % FBS in PBS. For surface cell analysis, cells were incubated with APC anti-mouse CD11b (OX-42; Biolegend, San Diego, CA, USA), PE anti-mouse CD45 (OX-1; Biolegend), APC anti-mouse CD4 (OX-35, BD Biosciences), and PE anti-mouse CD8a (OX-8, BD Biosciences) for 30 min at 4 °C. Microglia and macrophages were identified based on their relative CD45 expression levels [54]. Briefly, after acquiring unstained and single color control samples to calculate compensation matrix, we acquired 1 × 10^4^ events within the combined gate based on physical parameters [forward scatter (FSC) and side scatter (SSC)]. CD11b^+^/CD45^+(low)^ cells and CD11b^+^/CD45^+(high)^ cells were gated as resident microglia and macrophages, respectively [54]. For intracellular cell analysis, cells were restimulated with phorbol-12-myristate-13-acetate and ionomycin and Golgistop in RPMI media. After 5 h, cells were stained with PerCP-Cy 5.5 anti-mouse CD4 (RM4-5; BD Biosciences), FITC anti-mouse IFN-γ (XMG1.2; BD Biosciences), PE anti-mouse IL-17A (TC11-18H10; BD Biosciences), PE anti-mouse IL-4 (11B11; BD Biosciences), PE anti-mouse CD25 (PC61.5; FJK-16 s; eBioscience), and APC anti-mouse/rat Foxp3 (FJK-16 s; eBioscience) (Additional file 2) for 30 min at 4 °C. The cells were washed twice with 2 % FBS in PBS and used for flow cytometry. To identify CD4^+^ T cell populations, we first gated on cells (1 × 10^4^) based on FSC and SSC properties. CD4^+^ T cells were used to analyze populations of Th1, Th2, Th17, and Treg cells on CD4. Three-color staining was performed for one cell for simultaneous analysis. Intracellular cytokine results were indicated as percentages within the CD4^+^ population. Data were collected on a FACS Calibur flow cytometer (BD Biosciences) and analyzed using Cell Quest Pro software (BD Biosciences).

### T cell culture and proliferation

Methods for T cell culture and proliferation are presented in Additional file 3.

### Co-culture of microglia with T cells

Primary mixed glia was isolated from brains of postnatal 1–3 day WT or *LysM-Cre/Ikkβ*^*F/F*^ mice. After removing meninges of brain, single-cells were cultured in DMEM containing 10 mM HEPES, 10 % FBS, 2 mM L-glutamine, and antibiotic/antimycotic in 75 cm^2^ flasks at 37 °C with 5 % CO_2_. Culture medium was changed every 2–3 days and glia cultured for 14 days. Detached microglial cells were incubated for 30 min. Non-adherent cells were removed. These cells were approximately 95 % pure based on CD11b^+^ flow cytometry analysis. At 15 days after EAE induction, 95 % pure CD4^+^ T cells were harvested from lymph node cells of WT and *LysM-Cre/Ikkβ*^*F/F*^ mice by anti-mouse CD4 magnetic beads (Miltenyil Biotec). CD4^+^ T cells (2 × 10^6^ cells/ml) were re-stimulated with MOG_35–55_ peptide (25 μg/ml) in the presence IL-2 and IL-12 (20 ng/ml, R&D Systems). After 7 days of culturing, surviving MOG_35–55_ peptide-specific T cells were co-cultured with microglia in DMEM containing 10 % FBS and MOG_35–55_ peptide (25 μg/ml). T cells were added to the microglia at an estimated ratio of 1:2 (0.5 × 10^5^ T cells: 1 × 10^5^ microglia). After 24 h, cells were harvested and subjected to T cell differentiation analysis using flow cytometry as described above.

### Evaluation of BBB disruption

The level of BBB disruption was detected by quantitative measurement for Evans blue content at the peak day of neurological impairment after immunization, as previously described [63]. Briefly, sterilized 2 % Evans blue solution was intravenously injected at a dose of 4.0 ml/kg per mouse (*n* = 5 per group). Thirty minutes after injection, the mouse was perfused with saline to remove the Evans blue dye in the vascular system. The brain and lumbar spinal cord were immediately removed, and images were captured. The brain and lumbar lesion of the spinal cord were homogenized with 2.5 ml PBS and mixed with 2.5 ml 60 % trichloroacetic acid to precipitate protein. The samples were centrifuged for 30 min at 1,000 g, and the supernatants were measured at 610 nm for the absorbance of Evans blue by using an enzyme-linked immunosorbent assay reader (Soft Max Pro-5; Molecular devices, CA, USA). The Evans blue content was expressed as micrograms per gram of brain and lumbar spinal cord. To histologically and immunohistochemically investigate BBB permeability, 2 h after Evans blue solution injection to EAE mice, spinal cords were harvested from each group (*n* = 5 per group) and fixed with 4 % paraformaldehyde solution. Cryo-sections in 30 μm thickness were prepared and used to evaluate extravasation of Evans Blue dye using confocal microscopy. Immunofluorescent staining was performed as described above (Immunohistochemical analysis).

### Induction of passive transferred EAE

Induction of transferred EAE was performed with published method [6, 43] with slight modifications. Briefly, T cells were isolated from lymph nodes of WT or *LysM-Cre/Ikkβ*^*F/F*^ donor 15–18 days after induction of active EAE and re-stimulated with MOG_35–55_ peptide (25 μg/ml) in the presence IL-2 and IL-12 (20 ng/ml, R&D Systems, Minneapolis, U.S.A.) in RPMI 1640 medium containing 10 % FBS and 1 % penicillin/streptomycin for 3 days. Purified T cells (1 × 10^7^) were transferred i.v. into sub-lethally irradiated WT or *LysM-Cre/Ikkβ*^*F/F*^ recipient mice. Disease development was daily monitored.

### Statistical analyses

Statistical analysis was performed using the SPSS 21.0 package (SPSS Inc, Chicago, USA) for Windows. Neurological scores obtained by EAE induction were analyzed using two-way analysis of variance (ANOVA) with repeated measures with one within-subjects factor (time) and two between-subject factors (WT and *LysM-Cre/Ikkβ*^*F/F*^ mice). The data from immunohistochemistry, Western blot, and PCR analysis were analyzed using one-way ANOVA with Tukey *post hoc* test for comparison of multiple groups. The data were presented as mean ± SEM. P values of less than 0.05 were accepted as statistically significant.

## Results

### Myeloid-specific *ikkβ* gene deletion regulates M1/M2 polarization of macrophages

**Fig. 1:**
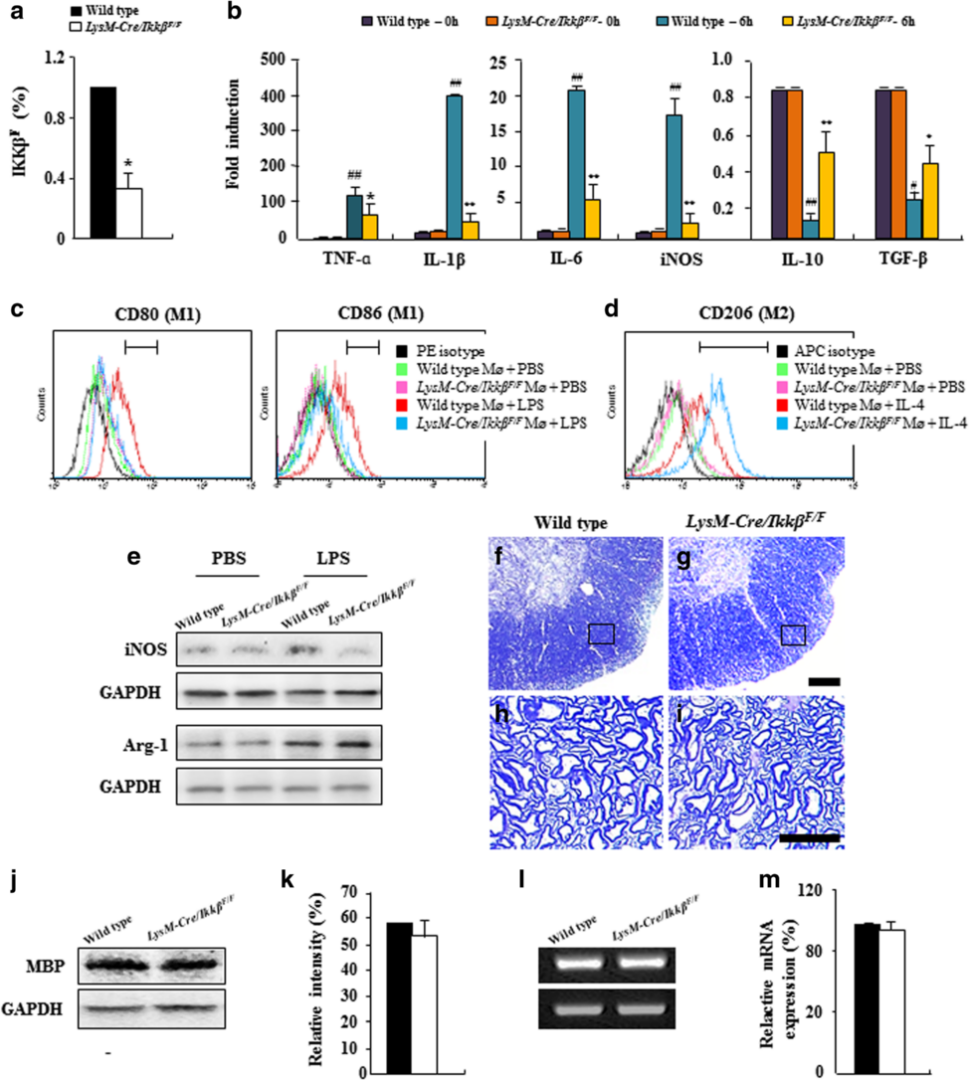
Myeloid cells of *LysM-Cre/Ikkβ* ^*F/F*^ mice are relatively refractory to M1-like proinflammatory activation. **a** CD11b^+^ cells were isolated from the lumbar spinal cord of adult WT and *LysM-Cre/Ikkβ* ^*F/F*^ mice using CD11b microbeads. Genomic DNA from CD11b^+^ cells of each group was analyzed by real-time RT-PCR to determine the rate of *ikkβ* deletion. **b** Macrophages obtained from normal WT and *LysM-Cre/Ikkβ* ^*F/F*^ mice were stimulated for 6 h with lipopolysaccharide. Macrophages were isolated and analyzed to evaluate the degree of activation of TNF-α, IL-1β, IL-6, iNOS, IL-10, and TGF-β with real-time RT-PCR. The levels of gene expressions were compared to normal control. **c**-**e** Macrophages derived from WT and *LysM-Cre/Ikkβ* ^*F/F*^ mice were treated with lipopolysaccharide or IL-4, incubated with PE anti-mouse CD80 (**c**), APC anti-mouse CD86 (**c**), and APC anti-mouse CD206 (**d**), and used for flow cytometry analysis (**c** and **d**). Alteration in protein expression of M1 (iNOS) and M2 marker (Arginine-1) was analyzed by Western blot analysis (**e**). **f**-**i** Semi-thin sections from the lumbar spinal cord of adult WT (F and H) and *LysM-Cre/Ikkβ* ^*F/F*^ mice (**g** and **i**) were stained with toluidine blue. Panels **h** and **i** display high magnification micrographs of sections in (panels **f** and **g**) marked with squares, respectively. Bars = 100 μm. **j**-**m** Spinal cord lysate obtained from adult WT and *LysM-Cre/Ikkβ* ^*F/F*^ mice was analyzed for the expression of MBP by immunoblotting (**j**) and were quantified (**k**). The same preparation was analyzed for mRNA expression of MBP by real-time RT-PCR (**l**) and was quantified (**m**). Data are representative of 3 independent experiments with similar results. (ANOVA test; **p < 0.01 and *p < 0.05 versus WT mice; #*p* < 0.05 and ##*p* < 0.01 versus normal control mice)

To investigate the in vivo function of proinflammatory macrophage/microglia activation in EAE, we used *LysM-Cre/Ikkβ*^*F/F*^ mice. We have previously demonstrated that the *ikkβ* gene was deleted specifically in peripheral macrophages and in brain microglia isolated from *LysM-Cre/Ikkβ*^*F/F*^ mice [9]. To study the effects of *ikkβ* deletion in EAE when demyelinating lesions are most prominent at the spinal cord level, we first confirmed the cell type-specific *ikkβ* gene deletion in spinal cord myeloid cells isolated from *LysM-Cre/Ikkβ*^*F/F*^ mice. In CD11b^+^ spinal cord myeloid cells isolated from the *LysM-Cre/Ikkβ*^*F/F*^ mice, expression of the IKKβ transcript was reduced by 70 % compared to WT mice (Fig. 1a). To test the effects of *ikkβ* deletion on the macrophage activation phenotype, we stimulated peritoneal macrophages derived from WT and *LysM-Cre/Ikkβ*^*F/F*^ mice with lipopolysaccharide, and then measured the gene expressions involved in inflammatory (M1) (TNF-α, IL-1β, IL-6, and iNOS) as well as alternative (M2) [IL-10 and transforming growth factor (TGF)-β] macrophage activation. Upon lipopolysaccharide stimulation for 6 h, TNF-α, IL- β, IL-6, and iNOS gene expressions were upregulated by 100.5 ± 0.5, 400 ± 0.7, 23.5 ± 0.4, and 19.7 ± 0.3 in the WT macrophages, respectively, compared to normal control (11.15 ± 0.65, 34.44 ± 15.21, 1.32 ± 0.59, and 1.11 ± 0.51) (Fig. 1b). In contrast, the induction levels of these M1 markers were reduced by more than 50 % in *LysM-Cre/Ikkβ*^*F/F*^ macrophages (Fig. 1b). To the contrary, lipopolysaccharide stimulation inhibited IL-10 and TGF-β expression by 18.5 ± 1.3 % and 31.4 ± 2.4 %, respectively, in WT macrophages, compared to normal control (Fig. 1b). However, the inhibition of these M2 markers was significantly ameliorated in *LysM-Cre/Ikkβ*^*F/F*^ macrophages. Gene expression levels of all inflammatory mediators in quiescent state (0 h) between WT and *LysM-Cre/Ikkβ*^*F/F*^ macrophages were comparable. In addition, we compared the populations of M1 (CD80- or CD86-positive) and M2 (CD206-positive) macrophages before and after lipopolysaccharide or IL-4 stimulation to understand M1/M2 polarization by flow cytometry analysis. As expected, the percentages of CD80- (3.5 ± 0.8 %; 572.0 ± 125.1 cells) and CD86-positive cells (2.9 ± 1.9 %; 221.0 ± 114.2 cells) in WT macrophages were significantly increased to 64.5 ± 8.5 % (4,917 ± 253.4 cells) and 43.9 ± 5.3 % (3,337 ± 521.3 cells), respectively, after lipopolysaccharide stimulation (Fig. 1c), while the percentage of CD206-positive cells (3.2 ± 1.3 %; 308 ± 143.4 cells) in *LysM-Cre/Ikkβ*^*F/F*^ macrophages was significantly increased to 84.3 ± 4.4 % (8,312 ± 281.3 cells) after IL-4 stimulation (Fig. 1d)*.* These results were in agreement with the alteration in protein expression level of M1 (iNOS) and M2 marker (Arginine-1) after lipopolysaccharide stimulation in WT or *LysM-Cre/Ikkβ*^*F/F*^ macrophages (Fig. 1e). As NF-κB signaling in Schwann cells is implicated in peripheral myelination [49], we examined whether *ikkβ* deletion in myeloid cells affected myelination in the spinal cord during development. Toluidine blue staining of lumbar spinal cord semi-thin sections displayed a similar pattern of myelination in the white matter of WT and *LysM-Cre/Ikkβ*^*F/F*^ mice (Fig. 1f-i). Likewise, the protein (Fig. 1j and k) and mRNA expression (Fig. 1l and m) of the myelin basic protein (MBP) as a marker for the degree of myelination in the spinal cord tissue were comparable (Fig. 1j-m).

### Myeloid-specific *ikkβ* gene deletion alleviates neurological impairment and spinal demyelination during EAE

**Fig. 2:**
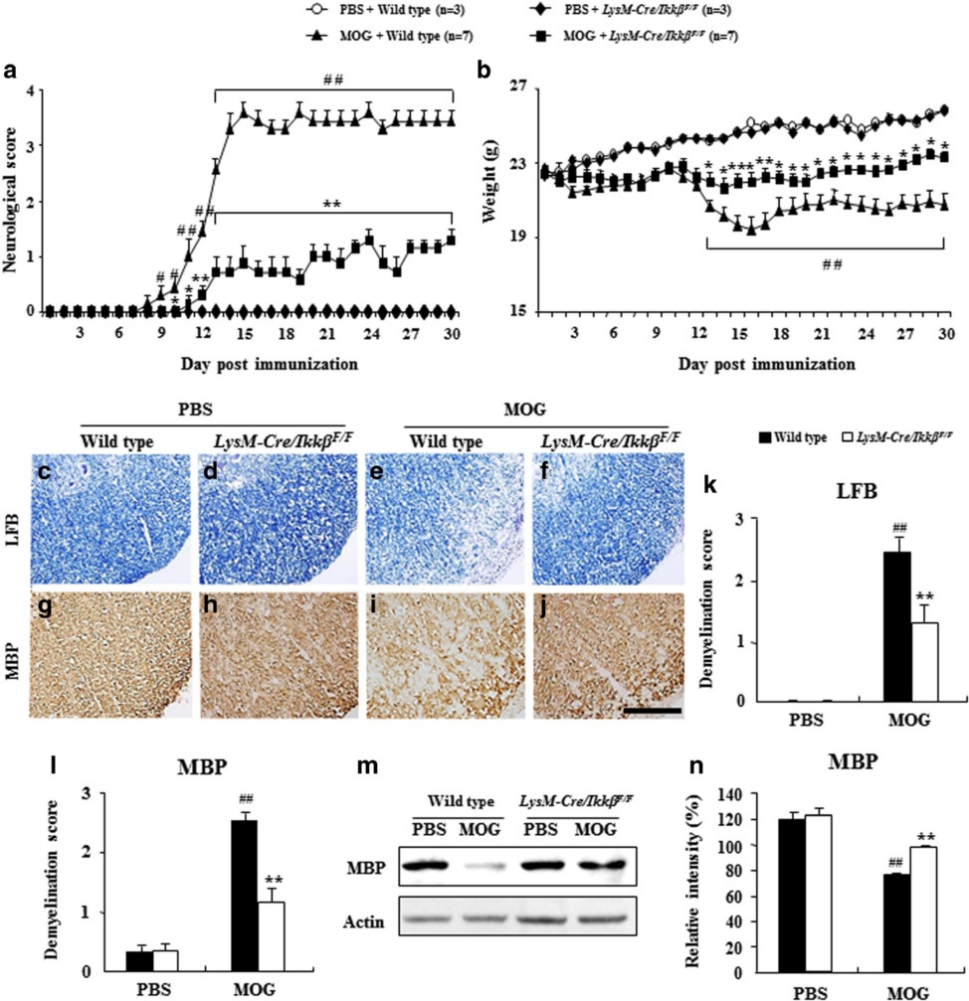
Myeloid-specific *ikkβ* gene deletion alleviates neurological impairment and demyelination in the spinal cord during EAE. **a** and **b** The clinical signs for all WT and *LysM-Cre/Ikkβ* ^*F/F*^ EAE mice were scored (**a**) and weighed (**b**) daily for 30 days. **c**-**l** Spinal cord sections were obtained from WT (**e**, **i**, **k**, and **l**) and *LysM-Cre/Ikkβ* ^*F/F*^ mice (**f**, **j**, **k**, and **l**) at 15–18 days after immunization, stained with luxol fast blue (**c**-**f**) or immunostained with MBP antibody (**g**-**j**), and analyzed to measure the degree of demyelination by immunization (**k** and **l**). Bars = 10 μm. **m** and **n** Spinal cord lysate obtained from WT and *LysM-Cre/Ikkβ* ^*F/F*^ mice at 15–18 days after immunization were analyzed for the expression of MBP by immunoblotting (**m**) and were quantified (**n**). Data are representative of 3 independent experiments with similar results. Data are expressed as mean clinical scores ± SEM. (ANOVA test; *p < 0.05 and **p < 0.01 versus WT EAE mice; #*p* < 0.05 and ##*p* < 0.01 versus normal control mice)

We then induced EAE in WT and *LysM-Cre/Ikkβ*^*F/F*^ mice by immunizing MOG_35–55_ peptide and monitored the clinical symptoms of EAE. MOG_35–55_ peptide immunization induced EAE-related symptoms in all the immunized WT and *LysM-Cre/Ikkβ*^*F/F*^ mice (7 out 7 mice) (Fig. 2a, b, and Table 1). In the WT mice, the first clinical signs appeared around day 7–8 (7.6 ± 0.7) and peaked around day 15 after immunization. However, *LysM-Cre/Ikkβ*^*F/F*^ mice had a remarkably delayed mean day of onset (10.2 ± 0.5) and ameliorated clinical symptoms (mean day of onset, maximal clinical score, sum of clinical score, and mortality) (Fig. 2a and Table 1). Mean body weight decreased negatively in correlation with the clinical score in the WT mice but was significantly increased in the *LysM-Cre/Ikkβ*^*F/F*^ mice (Fig. 2b). Sections of lumbar spinal cord prepared from WT and *LysM-Cre/Ikkβ*^*F/F*^ mice at the peak clinical signs (around day 15–18 after immunization) were stained with luxol fast blue (LFB) to examine whether the severity of clinical signs is correlated with the level of spinal demyelination. In the EAE-induced WT mice, LFB-negative areas representing demyelinated lesions appeared in the ventral spinal cords. However, the level of demyelination was markedly reduced in the *LysM-Cre/Ikkβ*^*F/F*^ mice (Fig. 2c-f and k). In accordance with the LFB data, demyelinated lesions were also detected by MBP immunostaining in the WT spinal cord after EAE induction. In *LysM-Cre/Ikkβ*^*F/F*^ spinal cord, however, MBP-negative lesion areas were not as obvious as in the WT spinal cord (Fig. 2g-j and l). In line with the immunohistochemical data, MBP protein expression was reduced by 37.3 % in EAE-induced WT spinal cord tissue compared to the sham-control, whereas it was reduced by only 11.3 % in the *LysM-Cre/Ikkβ*^*F/F*^ mice (Fig. 2m and n).

**Table 1:** Myeloid-specific *ikkβ* gene deletion ameliorates a subsequent encephalogenic challenge

	Group	Incidence (%)	Mean day of onset (± SEM)	Maximal clinical score (± SEM)	Sun of clinical score (± SEM)	Mortality (%)
PBS	Wild type	3/3	0.0 ± 0.0	0.0 ± 0.0	0.0 ± 0.0	0
	*LysM-Cre/Ikkβ* ^*F/F*^	3/3	0.0 ± 0.0	0.0 ± 0.0	0.0 ± 0.0	0
MOG	Wild type	7/7	7.6 ± 0.7	3.4 ± 0.2^#^	64.1 ± 1.7^#^	0
	*LysM-Cre/Ikkβ* ^*F/F*^	7/7	10.2 ± 0.5	2.6 ± 0.2*	17.3 ± 3.0*	0

ANOVA test; *p < 0.05 versus WT EAE mice; #*p* < 0.05 versus normal control mice

### Myeloid-specific *ikkβ* gene deletion inhibits the recruitment/infiltration of microglia and macrophages in spinal cord lesion during EAE

**Fig. 3:**
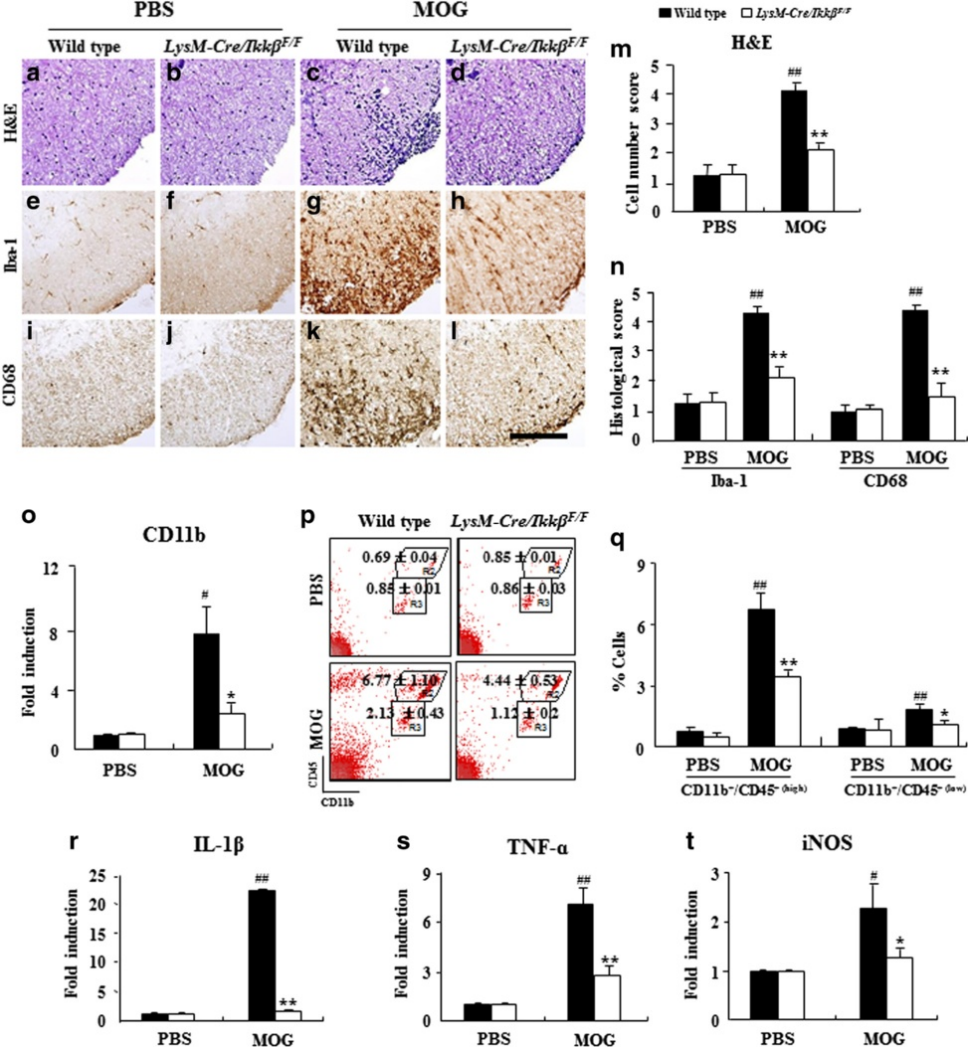
Myeloid-specific *ikkβ* gene deletion inhibits the recruitment/infiltration of residential microglia and of peripheral macrophages in spinal cord lesion during EAE. **a**-**n** Spinal cord sections were obtained from WT and *LysM-Cre/Ikkβ* ^*F/F*^ mice (*n* = 5) at 15–18 days after immunization, stained with H&E dye (**a**-**d**), and immunostained with anti-Iba-1 (**e**-**h**) and anti-CD68 antibodies (**i**-**l**) to investigate the degree of cellular recruitment/infiltration. Representative photographs show ventrolateral white matter of the lumbar spinal cord (**a**-**l**). The degree of recruitment/infiltration was quantified and the data are expressed as mean score ± SEM (**m** and **n**). Bars = 100 μm. **o** Spinal cords from each group (*n* = 5) at 15–18 days after immunization were analyzed with real-time RT-PCR. Data are expressed as mean fold induction ± SEM. **p** and **q** Spinal cords were dissected from each group (*n* = 5) at 15–18 days after immunization to investigate the degree of recruitment/infiltration of macrophages with flow cytometry. Tissues were dissociated, and cells were incubated with APC-conjugated anti-CD11b and PE-conjugated anti-CD45 antibodies. CD11b^+^ cells were divided into CD11b^+^/CD45^+(high)^ cells (R2; macrophage) and CD11b^+^/CD45^+(low)^ cells (R3; microglia) populations based on CD45 expression levels (**p**), and the percentages of each population are denoted in the graph (**q**). Mean ± SEM values from 3 independent experiments are shown in the graph. **r**-**t** Total RNA was isolated from the lumbar spinal cord of each group (*n* = 3) at day 15–18 post-immunization to measure the level of the mRNA expression of IL-1β (**r**), TNF-α (**s**), and iNOS (**t**) with real-time RT-PCR. The mRNA level of each gene is presented as the fold induction ± SEM compared with the levels measured in the normal control mice. (ANOVA test; *p < 0.05 and **p < 0.01 versus WT EAE mice; #*p* < 0.05 and ##*p* < 0.01 versus normal control mice)

We then assessed whether the reduced clinical signs and demyelination in *LysM-Cre/Ikkβ*^*F/F*^ mice was associated with the level of activation and recruitment of immune cells into demyelinated lesions by H&E staining. Consistent with the severity level in clinical symptoms and demyelination, immune cell infiltration on the peak day markedly increased in the white matter of the spinal cords of EAE-induced WT mice compared to sham-control mice (Fig. 3a-d and m). Inflammatory cells were detected adjacent to the subarachnoid space, within the parenchyma in perivascular lesions, and in the spinal cord of WT mice. However, their distribution was significantly restricted in the spinal cord of *LysM-Cre/Ikkβ*^*F/F*^ mice (Fig. 3a-d and m). Similarly, the EAE-induced microglia activation and macrophage infiltration measured by ionized calcium-binding adapter molecule-1 (Iba-1) and CD68-immunoreactivity, respectively, were substantially reduced in the spinal cords of *LysM-Cre/Ikkβ*^*F/F*^ mice (Fig. 3e-l and n). In agreement with the histological data, mRNA expression of CD11b (a marker of myeloid cells) was also up-regulated in the spinal cord of WT mice by EAE induction, while the induction level was reduced by more than 80 % in the spinal cords of *LysM-Cre/Ikkβ*^*F/F*^ mice (Fig. 3o). Since there are limitations with differentiating macrophages and microglia based on immunohistochemistry, we further characterized microglia vs. macrophage populations by flow cytometry. Upon immunization, the percentage of CD11b^+^/CD45^+(low)^ cells (R3 rectangle in Fig. 3p) representing brain resident microglia [9, 23, 33, 34] was increased to 2.13 ± 0.43 % in the WT spinal cords, whereas it was increased to 1.12 ± 0.20 % in *LysM-Cre/Ikkβ*^*F/F*^ spinal cords (Fig. 3p and q). The percentage of CD11b^+^/CD45^+(high)^ cells (R2 rectangle in Fig. 3p) representing macrophages was also increased to 6.77 ± 1.10 % in the WT spinal cords, whereas it was increased to only 4.44 ± 0.53 % in *LysM-Cre/Ikkβ*^*F/F*^ spinal cords (Fig. 3p and q). To confirm the effect of myeloid *ikkβ* deletion in curtailing the M1-like proinflammatory macrophage/microglia activation in the spinal cord in vivo, we compared the expression levels of M1 marker genes (IL-1β, TNF-α, and iNOS) between the lumbar spinal cords of WT and *LysM-Cre/Ikkβ*^*F/F*^ mice 15–18 days after immunization (Fig. 3r-t). In the spinal cord of WT mice, upon EAE induction, the mRNA expression of IL-1β, TNF-α, and iNOS were increased by 22.3 ± 0.3, 7.1 ± 1.1, and 2.3 ± 2.1, respectively. The EAE-induced IL-1β expression was almost completely blocked in the *LysM-Cre/Ikkβ*^*F/F*^ mice (Fig. 3r). TNF-α and iNOS expressions were also substantially compromised in the *LysM-Cre/Ikkβ*^*F/F*^ mice (Fig. 3s and t).

### Myeloid-specific *ikkβ* gene deletion decreases the size of lymphatic organs and inhibits the differentiation/proliferation of CD4^+^ T cells during EAE

**Fig. 4:**
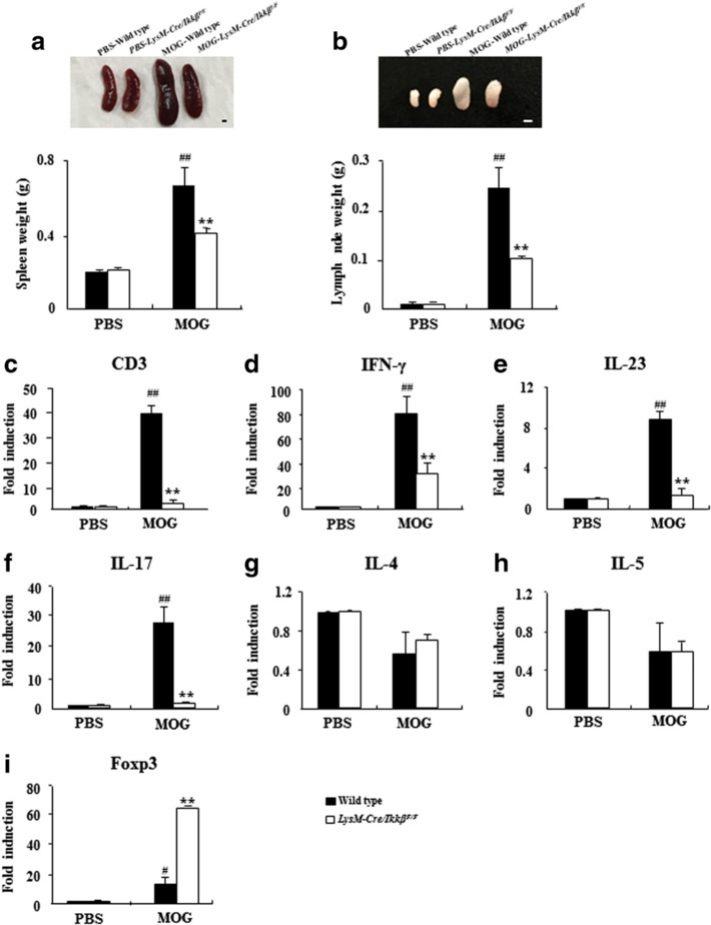
Myeloid-specific *ikkβ* gene deletion decreases the size of lymphatic organs and regulates transcription factors associated with Th1, Th17, and Treg cells. **a** and **b** The spleen (**a**) and lymph nodes (**b**) were dissected from each group (*n* = 5) at 15–18 days after immunization and weighted. Bars = 100 mm. **c** Spinal cords were dissected from each group (*n* = 5) at 15–18 days after immunization to investigate the degree of recruitment/infiltration of T cells with real-time RT-PCR. The degree of mRNA expression of CD3 was expressed as mean fold-induction ± SEM. **d**-**i** Total RNA was isolated from the lumbar spinal cords of each group (*n* = 3) at days 15–18 post-immunization to measure the level of the mRNA expression of inflammatory mediators with real-time RT-PCR. The mRNA levels of IFN-γ (**d**), IL-23 (**e**), IL-17 (**f**), IL-4 (**g**), IL-5 (**h**), and Foxp3 (**i**) are presented as the fold induction compared with the levels measured in the normal control mice. Data are represented as mean ± SEM. (ANOVA test; **p < 0.01 versus WT EAE mice; #*p* < 0.05 and #*p* <0.01 versus normal control mice)

Since hypertrophy of spleen and lymph nodes are observed in EAE mice [46], we measured weight to compare their sizes at 15–18 days after immunization. As expected, their weights were increased by immunization. The increase in weights of spleen (0.38 ± 0.01 g) and lymph nodes (0.10 ± 0.01 g) of *LysM-Cre/Ikkβ*^*F/F*^ mice were significantly lower than that of WT mice (0.68 ± 0.04 g in spleen and 0.25 ± 0.05 g in lymph nodes; Fig. 4a and b). Activation and recruitment of auto-reactive T cells are critical initiators and mediators of EAE pathogenesis [22]. To investigate the mechanisms underlying the ameliorated EAE pathogenesis in the *LysM-Cre/Ikkβ*^*F/F*^ mice, we examined the extent of T cells infiltration into the spinal cord of WT and *LysM-Cre/Ikkβ*^*F/F*^ mice after EAE induction. The mRNA expression of CD3, as a marker for T cells, was increased by 40-fold in the spinal cord of WT mice on days 15–18 of EAE, which was almost completely blocked in *LysM-Cre/Ikkβ*^*F/F*^ mice (Fig. 4c). Characterization of the T cell subtypes by flow cytometry showed that the percentage of CD4^+^ T cells was markedly increased after EAE induction in the spinal cord (14.47 ± 1.21 %; Table 2 and Additional file 4a) and lymph nodes (27.77 ± 2.33 %; Table 2 and Additional file 4b) of WT mice. However, the increase in CD4^+^ Th cells was significantly diminished in *LysM-Cre/Ikkβ*^*F/F*^ EAE mice both in the spinal cord (8.18 ± 0.53 %; Table 2 and Additional file 4a) and lymph nodes (19.96 ± 2.96 %; Table 2 and Additional file 4b). The percentage of cytotoxic CD8^+^ T cells was not significantly affected by immunization or myeloid IKKβ deletion (Table 2 and Additional file 4a and b). In naïve mice, the percentages of CD4^+^ T cells and CD8^+^ T cells in the spinal cords (Table 2 and Additional file 4a) and lymph nodes (Table 2 and Additional file 4b) were comparable in WT and *LysM-Cre/Ikkβ*^*F/F*^ mice.

**Table 2:** Myeloid-specific *ikkβ* gene deletion regulates the percentages of CD4^+^, CD4^+^/IFN-γ^+^, CD4^+^/IL-17^+^, and CD4^+^/CD25^+^/Foxp3^+^ T cells

A				
Spinal cord	PBS		MOG	
	Wild type	LysM-Cre/Ikkβ^F/F^	Wild type	LysM-Cre/Ikkβ^F/F^
CD4^+^	0.52 ± 0.17	0.65 ± 0.21	14.47 ± 1.21^##^	8.18 ± 0.53^**^
CD8^+^	2.53 ± 1.38	2.89 ± 0.13	3.07 ± 0.74	2.76 ± 0.06
CD4^+^/IFN-γ^+^	2.97 ± 0.04	2.98 ± 0.95	11.33 ± 0.69^##^	6.34 ± 0.22^**^
CD4^+^/IL-17^+^	3.84 ± 0.94	3.01 ± 0.12	14.35 ± 1.21^##^	5.43 ± 0.63^**^
CD4^+^/IL-4^+^	0.41 ± 0.09	0.49 ± 0.29	0.87 ± 0.13	0.94 ± 0.27
CD4^+^/Foxp3^+^	0.31 ± 0.45	0.37 ± 0.57	4.59 ± 1.97^#^	13.49 ± 3.21^*^
CD4^+^/CD25^+^/Foxp3^+^	0.46 ± 0.06	0.74 ± 0.10	1.55 ± 0.15^##^	4.05 ± 0.80^*^
B				
Lymph node	PBS		MOG	
	Wild type	*LysM-Cre/Ikkβ* ^*F/F*^	Wild type	*LysM-Cre/Ikkβ* ^*F/F*^
CD4^+^	8.43 ± 0.64	8.13 ± 0.84	27.77 ± 2.33^##^	19.96 ± 2.96^*^
CD8^+^	4.85 ± 0.00	4.39 ± 0.10	4.71 ± 1.08	4.06 ± 0.72
CD4^+^/IFN-γ^+^	1.01 ± 0.09	1.24 ± 0.01	9.40 ± 1.98^##^	4.59 ± 0.72^*^
CD4^+^/IL-17^+^	0.13 ± 0.53	0.10 ± 0.34	14.76 ± 2.64^##^	4.13 ± 0.66^**^
CD4^+^/IL-4^+^	4.55 ± 0.60	4.29 ± 0.52	3.49 ± 1.32^##^	4.01 ± 0.94
CD4^+^/Foxp3^+^	16.49 ± 1.02	15.21 ± 0.37	8.01 ± 1.31^#^	11.31 ± 0.20^*^
CD4^+^/CD25^+^/Foxp3^+^	5.00 ± 0.21	4.96 ± 0.57	2.75 ± 0.01^##^	4.41 ± 0.13^**^

Spinal cords (A) and lymph nodes (B) were dissected from each group (*n* = 5) at day 15–18 post-immunization to investigate the degree of differentiation and recruitment/infiltration of CD4^+^ T cells with flow cytometry. Spot plot graphs and their legends in details can be found in the Additional file 4. Data are represented as mean ± SEM. (ANOVA test; * *p* < 0.05 and **p < 0.01 versus WT EAE mice; #*p* < 0.05 and ##*p* <0.01 versus normal control mice)

### Myeloid-specific *ikkβ* gene deletion decreases the percentages of CD4^+^ T, Th1, and Th17 cells but increases the percentage of Treg cells during EAE

Since naïve CD4 T cells may differentiate into Th1, Th2, Th17, and regulatory T (Treg) cells during T cell receptor (TCR) activation in a particular cytokine milieu involved in autoimmunity, we characterized each subtype of CD4^+^ T cells in the EAE-induced spinal cord. It is well-known that MS/EAE is mediated by encephalitogenic Th1 and Th17 cells that produce proinflammatory cytokines such as IFN-γ and IL-17, respectively [12, 59]. Thus, we investigated the effects of myeloid IKKβ deletion on the infiltration/activation of CD4^+^/IFN-γ^+^ (Th1) and CD4^+^/IL-17^+^ T (Th17) cells in the spinal cords and lymph nodes. In the EAE-induced mice, the CD4^+^/IFN-γ^+^ Th1 population increased to 11.33 ± 0.69 % and 9.40 ± 1.98 % in the spinal cord (Table 2 and Additional file 4a) and lymph node (Table 2 and Additional file 4b), respectively. Similarly, CD4^+^/IL-17^+^ T cell populations in the spinal cord and lymph node increased to 14.35 ± 1.21 % (Table 2 and Additional file 4a) and 14.76 ± 2.64 % (Table 2 and Additional file 4b), respectively. However, in *LysM-Cre/Ikkβ*^*F/F*^ mice, Th1 and Th17 cells increased to only 6.34 ± 0.22 % and 5.43 ± 0.63 %, respectively, in the spinal cord (Table 2 and Additional file 4a) and to 4.59 ± 0.72 % and 4.13 ± 0.66 %, respectively, in the lymph nodes (Table 2 and Additional file 4b). Additionally, mRNA induction of IFN-γ, IL-23 (an interleukin that induces differentiation of naïve Th cells to Th17 cells), and IL-17 in the EAE-induced spinal cord was markedly reduced by myeloid IKKβ deletion (Fig. 4d-f). The percentage of CD4^+^/IL-4^+^ Th2 cells in the spinal cords (Table 2 and Additional file 4a) or lymph nodes (Table 2 and Additional file 4b) was not significantly altered in WT and *LysM-Cre/Ikkβ*^*F/F*^ mice upon EAE induction. Likewise, mRNA expressions of IL-4 (a cytokine that induces the differentiation of naïve Th cells to Th2 cells) and IL-5 (an interleukin produced by Th2 cells) were not significantly affected by immunization or myeloid IKKβ deletion (Fig. 4g and h). Interestingly, the number of CD4^+^/Foxp3^+^ and CD4^+^/CD25^+^/Foxp3^+^ Treg cells that maintain tolerance to self-antigens and suppress autoimmune responses increased in the spinal cord of EAE-induced mice compared to normal control mice (0.46 ± 0.06 % vs. 1.55 ± 0.15 %) (Table 2 and Additional file 4a). Notably, this increase was further potentiated by myeloid IKKβ deletion (0.47 ± 0.10 % vs. 4.05 ± 0.80 %), which corresponded with mRNA expression of Foxp3 (a “master gene” for controlling the development and function of Treg cells) in the spinal cord (Fig. 4i). Although the Treg cell populations in the lymph node were reduced in EAE-induced mice compared to the sham-control, there were still more Treg cells in the *LysM-Cre/Ikkβ*^*F/F*^ mice compared to the WT mice (Table 2 and Additional file 4b).

### Myeloid-specific *ikkβ* gene deletion inhibits the activity of co-stimulatory factors in macrophages and regulates the differentiation/proliferation of T cells by co-culture with microglia

**Fig. 5:**
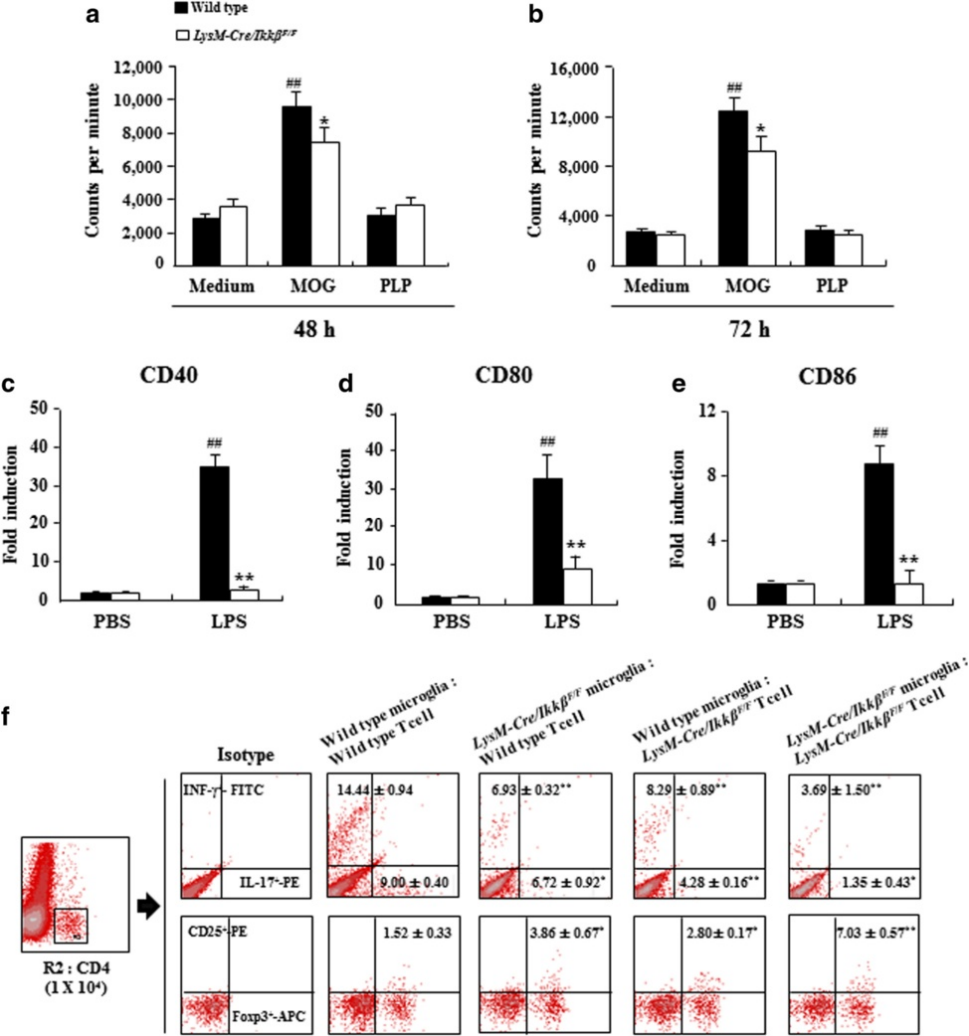
Myeloid-specific *ikkβ* gene deletion inhibits the mRNA expression of co-stimulatory molecules in macrophages and regulates the proliferation of T cells by co-culture with microglia. **a**-**b** Spleen mononuclear cells obtained from WT and *LysM-Cre/Ikkβ* ^*F/F*^ mice at 15–18 days after immunization were incubated with MOG_35–55_ peptide and PLP peptide for 48 and 72 h. The cells were pulsed for 18 h with solution containing ^3^H-methylthymidine and thymidine incorporation was measured. The results were expressed as counts per minute (mean ± SEM). **c**-**e** Macrophages obtained from WT and *LysM-Cre/Ikkβ* ^*F/F*^ mice were stimulated with lipopolysaccharide for 6 h. The mRNA expression levels of CD40 (**c**), CD80 (**d**), and CD86 (**e**) were determined using real-time RT-PCR. Data are represented as mean ± SEM. (ANOVA test; *p < 0.05 and **p < 0.01 versus supernatant from WT mice; ##*p* < 0.01 versus supernatant from normal control mice). **f** Microglial cells were isolated from brains of postnatal 1–3 day WT or *LysM-Cre/Ikkβ* ^*F/F*^ mice. At 15 days after EAE induction, CD4^+^ T cells (95 % pure) were harvested from lymph nodes of each group using anti-mouse CD4 magnetic beads. MOG_35–55_ peptide-specific T cells were co-cultured with microglia for 24 h and incubated with APC anti-mouse CD4, PE anti-mouse IFN-γ, FITC anti-mouse IL-17A, PE anti-mouse CD25, and APC anti-mouse Foxp3 antibodies. Far left, CD4^+^ T cell gate (1 × 10^4^ cells in R2) used to identify CD4^+^/IFN-γ^+^, CD4^+^/IL-17^+^, and CD4^+^/CD25^+^/Foxp3^+^ T cells. Representative data and mean ± SEM values from 3 independent experiments are shown in spot plot graphs. (ANOVA test; *p < 0.05 and **p < 0.01 versus WT microglia and WT T cell group)

We next investigated the direct effect of myeloid cell specific IKKβ deletion on antigen-specific T cell proliferation in EAE. When spleen mononuclear cells from WT mice were incubated for 48 or 72 h with MOG_35–53_ peptide, significant splenocyte proliferation, a measure of autoreactive T cell proliferation, was detected in WT mice, but was markedly diminished in splenocytes prepared from *LysM-Cre/Ikkβ*^*F/F*^ mice (Fig. 5a and b). The proteolipid (PLP) peptide failed to induce T cell proliferation in both WT and *LysM-Cre/Ikkβ*^*F/F*^ spleen cells, indicating MOG_35–55_ peptide-specific autoreactive T cell proliferation (Fig. 5a and b). T cell activations are regulated by co-stimulatory signals from antigen presenting cells [17]. To test whether the diminished auto-reactive T cell proliferation observed in *LysM-Cre/Ikkβ*^*F/F*^ splenocyte culture was due to reduced co-stimulatory signals from the IKKβ-deleted myeloid cells, we tested the expression of co-stimulatory molecules on WT and *LysM-Cre/Ikkβ*^*F/F*^ macrophages. Upon lipopolysaccharide stimulation, the CD40, CD80, and CD86 transcripts were induced in WT macrophages by 35.7 ± 2.5, 32.9 ± 6.1, and 8.8 ± 0.9, respectively (Fig. 5c-e). However, lipopolysaccharide-stimulated costimulatory molecule expression was significantly reduced in macrophages from *LysM-Cre/Ikkβ*^*F/F*^ mice (Fig. 5c-e), which might contribute to the reduced autoreactive T cell responses in the *LysM-Cre/Ikkβ*^*F/F*^ mice. Nevertheless, it is hard to understand whether these were cause or consequence phenomenon and how deletion of IKKβ in myeloid cells might have induced Th1, Th17, and Treg cells. Thus, we performed co-culture experiments of microglia and CD4^+^ T cells derived from WT and *LysM-Cre/Ikkβ*^*F/F*^ mice. The percentages (6.93 ± 0.32 % and 6.72 ± 0.92 %) of CD4^+^/IFN-γ^+^ and CD4^+^/IL-17^+^ cells in the co-culture group of *LysM-Cre/Ikkβ*^*F/F*^ microglia and WT CD4^+^ T cells were lower than that in the co-culture group of WT microglia and WT CD4^+^ T cells (14.44 ± 0.94 % and 9.00 ± 0.40 %), while the percentage (3.86 ± 0.67 %) of CD4^+^/CD25^+^/Foxp3^+^ cells in the co-culture group of *LysM-Cre/Ikkβ*^*F/F*^ microglia and WT CD4^+^ T cells was higher than that in the co-culture group of WT microglia and WT CD4^+^ T cells (1.52 ± 0.33 %) (Fig. 5f). The percentages (3.69 ± 1.50 % and 1.35 ± 0.43 %) of CD4^+^/IFN-γ^+^ and CD4^+^/IL-17^+^ cells in the co-culture group of *LysM-Cre/Ikkβ*^*F/F*^ microglia and *LysM-Cre/Ikkβ*^*F/F*^ CD4^+^ T cells were lower than that in the co-culture group of *LysM-Cre/Ikkβ*^*F/F*^ microglia and WT CD4^+^ T cells. And the percentage (7.03 ± 0.57 %) of CD4^+^/CD25^+^/Foxp3^+^ cells in the co-culture group of *LysM-Cre/Ikkβ*^*F/F*^ microglia and *LysM-Cre/Ikkβ*^*F/F*^ CD4^+^ T cells was higher than that in the co-culture group of *LysM-Cre/Ikkβ*^*F/F*^ microglia and WT CD4^+^ T cells (Fig. 5f).

### Myeloid-specific *ikkβ* gene deletion inhibits BBB permeability with during EAE

**Fig. 6:**
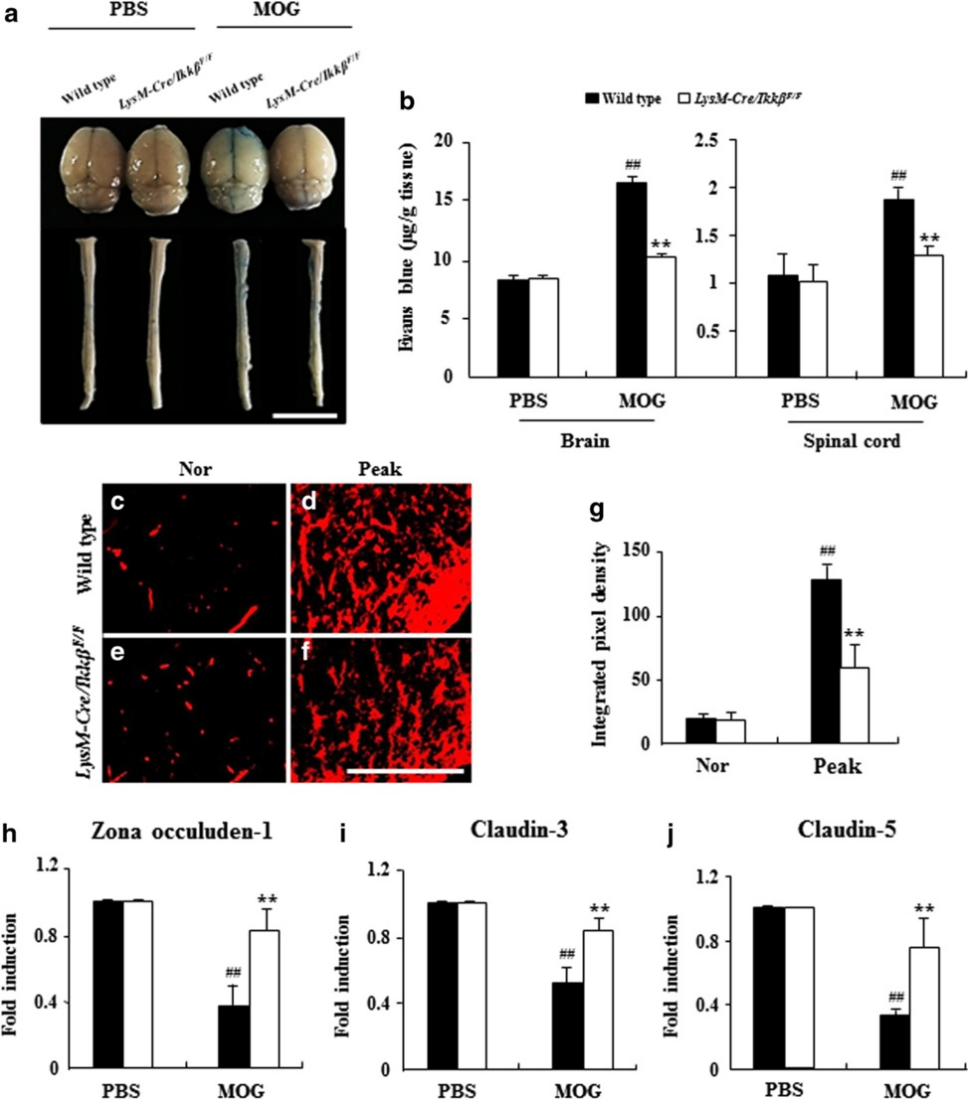
Myeloid-specific *ikkβ* gene deletion inhibits BBB permeability in the spinal cord with during EAE. **a** and **b** The amount of extravasated Evans blue dye was quantified in the brain and lumbar spinal cord, at 15–18 days after immunization (*n* = 5 per group). Representative pictures (**a**) and quantitative data (**b**) are displayed. *Blue* color in A indicates extravasated Evans blue dye from blood vessels. **c**-**g** Representative confocal images of extravasated Evans blue dye in the lumbar spinal cord from each group (*n* = 5; **c**-**f**) and quantified data (**g**). *Red* color in the (**c**-**f**) indicates extravasated Evans blue dye from blood vessels. **h**-**j** The mRNA expression levels of junctional proteins [zona occuluden-1 (**h**), claudin-3 (**i**), and claudin-5 (**j**)] in spinal cords from each group were analyzed by real-time RT-PCR. Data are representative of 3 independent experiments with similar results and the data are expressed as mean fold induction ± SEM. ANOVA test; **p < 0.01 versus supernatant from WT mice; ##*p* < 0.01 versus supernatant from normal control mice

Besides auto-reactive T cell activation, blood brain barrier (BBB) compromise is a pathological hallmark of EAE pathogenesis [1, 2, 14, 31, 42]. Consequently, we characterized the EAE-induced BBB disruption in WT and *LysM-Cre/Ikkβ*^*F/F*^ mice by Evans blue leakage on the peak day of EAE clinical signs. The macroscopic images of the brain and spinal cord showed that the degree of Evans blue leakage was significantly reduced in *LysM-Cre/Ikkβ*^*F/F*^ mice (Fig. 6a). For quantification, the Evans blue content was increased up to 16.6 ± 0.5 mg/g in the brain and to 1.9 ± 0.1 mg/g in the lumbar spinal cord of the WT EAE mice compared with the baseline level of the sham-control mice, whereas the Evans blue contents in the *LysM-Cre/Ikkβ*^*F/F*^ mice were only 10.1 ± 0.1 mg/g in the brain and 1.3 ± 0.1 mg/g in the lumbar spinal cord (Fig. 6b). In agreement with these results, confocal microscopic images (Fig. 6c-f) and their quantified graph (Fig. 6g) showed that the intensity of Evans blue dye was increased by 134.56 ± 0.36 pixels in the white matter of lumbar spinal cord of the WT EAE mice compared to the baseline level of the sham-control mice 33.45 ± 8.22 pixels), whereas the intensity in the *LysM-Cre/Ikkβ*^*F/F*^ mice were only 63.73 ± 0.45 pixels in the white matter of lumbar spinal cord (Fig. 6c-g). To investigate the mechanisms underlying the reduced BBB compromise observed in the *LysM-Cre/Ikkβ*^*F/F*^ mice, we examined the expressions of genes involved in tight junction formation in the cerebral endothelial cells such as zona occludens and claudin [52]. Upon EAE induction, the mRNA expression of zona occludens-1, claudin-3, and claudin-5 decreased in the injured spinal cord of WT mice (Fig. 6h-j), indicating a compromise of the tight junction formation after EAE. However, the decrease in the tight junction components was significantly ameliorated in the *LysM-Cre/Ikkβ*^*F/F*^ mice.

### Myeloid-specific *ikkβ* gene deletion maintains BBB integrity in the spinal cord during EAE

**Fig. 7:**
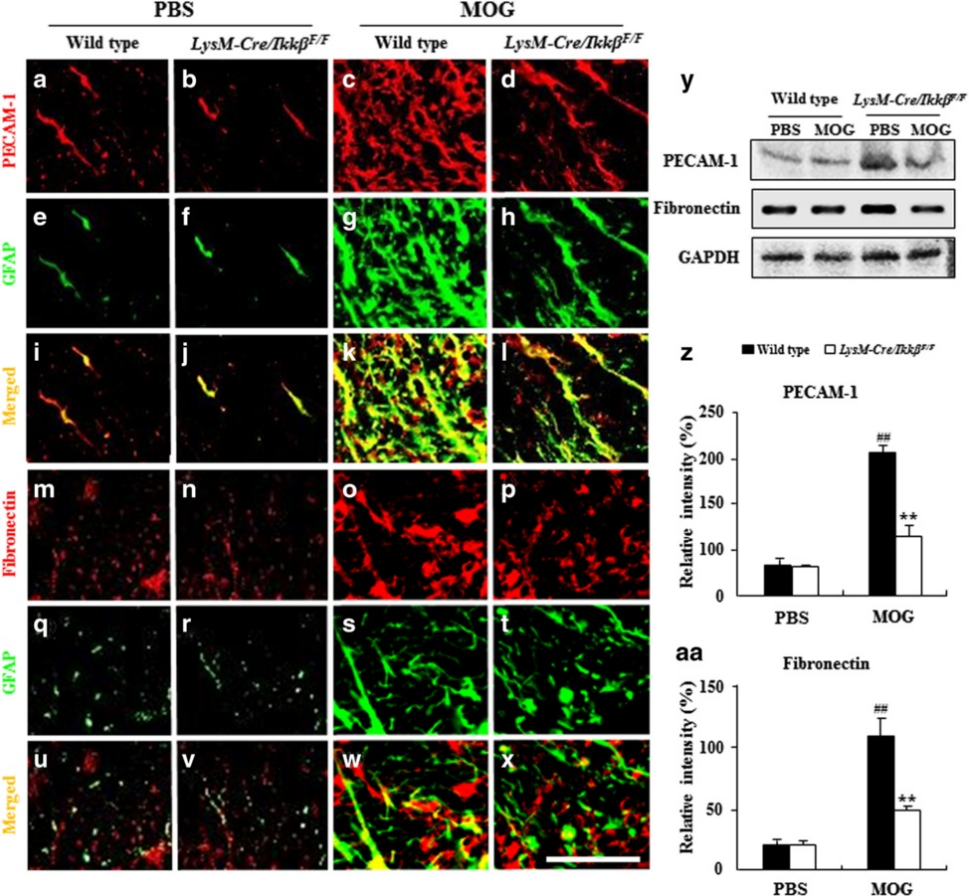
Myeloid-specific *ikkβ* gene deletion reduces disruption of BBB integrity in the spinal cord during EAE. **a**-**x** Spinal cord sections from WT and *LysM-Cre/Ikkβ* ^*F/F*^ EAE mice (*n* = 5 per group) were double immunostained with anti-PECAM-1 (**a**-**d**), fibronectin (**m**-**p**), or GFAP (**e**-**h** and **q**-**t**) antibodies at 15–18 days after immunization. Bars = 10 μm. **y**-**aa** Spinal cords from each group (*n* = 3 per group) were analyzed to test the level of protein expression of PECAM-1 (**y** and **z**) and fibronectin (**y** and **aa**) with Western blot. Data are represented as mean ± SEM. (ANOVA test; **p < 0.01 versus WT EAE mice; ##*p* < 0.01 versus normal control mice)

In addition, we characterized the expression of platelet endothelial cell adhesion molecule-1 (PECAM-1, CD31) and fibronectin in astrocytes. These are indicators of BBB disruption [15, 16, 27]. In EAE-induced WT spinal cord, immunofluorescence for PECAM-1 (Fig. 7a-l) and fibronectin (Fig. 7m-x) was elevated around or near the activated GFAP-positive astrocytes. Some immunofluorescence for PECAM-1 and fibronectin was also detected in the activated GFAP-positive astrocytes in the white matter of the spinal cords from the WT mice compared to those from the normal control group. However, the increased expressions were clearly decreased, corresponding to the suppression of astrocyte activation in the *LysM-Cre/Ikkβ*^*F/F*^ EAE mice (Fig. 7d, h, l, p, t, and x). The levels of PECAM-1 and fibronectin expression in the lumbar spinal cord measured by Western blot assay were consistent with the immunohistochemistry data (Fig. 7y-aa). To further understand the association between conditional deletion of IKKβ in myeloid cells and immune-mediated reactions/BBB permeability, we compared the expression of PECAM-1 and Evans blue dye at a different time points (onset and peak stages) during EAE (Additional file 5). In EAE-induced WT spinal cord, immunofluorescence signals for PECAM-1 (Additional file 5a-i) and Evans blue dye (Additional file 5a-c) were slightly increased at the onset stage of neurological impairment compared to those in normal control mice. When the signals were merged with each other, their intensities were slightly decreased in EAE-induced *LysM-Cre/Ikkβ*^*F/F*^ spinal cord (Additional file 5a-d and a-k). However, the immunofluorescence intensities of PECAM-1 (Additional file 5a-k) and Evans blue dye (Additional file 5a-e) were clearly increased in the astrocyte of the spinal cord at the peak stage of neurological impairment (15–18 days post-immunization) in EAE-induced WT mice compared to those in normal control mice. However, these intensities were decreased in EAE-induced *LysM-Cre/Ikkβ*^*F/F*^ spinal cord (Additional file 5a-f, l, and r). The results were in agreement with changed in the level of Iba-1- (Additional file 5b) and CD68-immunoreactivity (Additional file 5c) in onset and peak stages of neurological impairment.

### Myeloid *IKK*β deletion attenuates transferred EAE

**Fig. 8:**
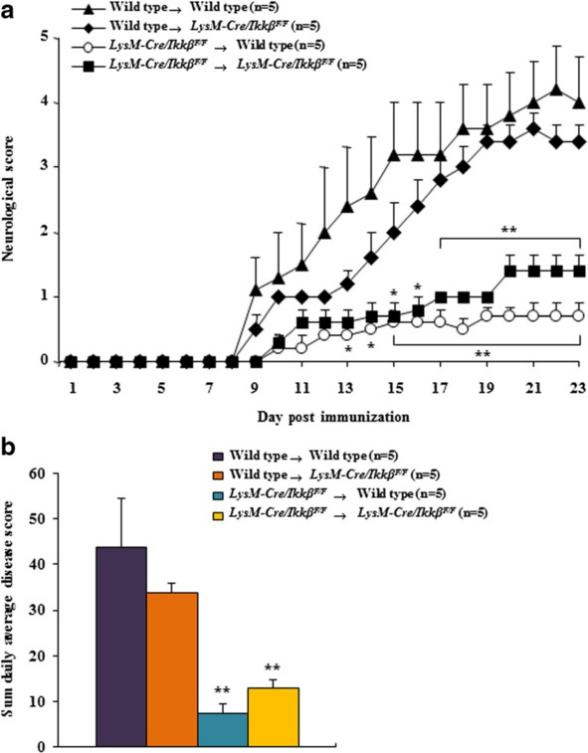
Myeloid-specific *ikkβ* gene deletion reduces transferred EAE. **a**-**b** T-cells were isolated from lymph nodes of WT or *LysM-Cre/Ikkβ* ^*F/F*^ donor (*n* = 5 per group) at 15–18 days after induction of active EAE and re-stimulated with MOG_35–55_ peptide (25 μg/ml). Purified T cells (1 × 10^7^) were transferred i.v. into sub-lethally irradiated WT or *LysM-Cre/Ikkβ* ^*F/F*^ recipient mice. Disease development was monitored daily (**a**) and cumulative disease index (**b**) was assessed. Experimental groups include Wild type → Wild type, Wild type → *LysM-Cre/Ikkβ* ^*F/F*^, *LysM-Cre/Ikkβ* ^*F/F*^ → Wild type, and *LysM-Cre/Ikkβ* ^*F/F*^ → *LysM-Cre/Ikkβ* ^*F/F*^. Data are represented as mean ± SEM. (ANOVA test; *p < 0.05 and **p < 0.01 versus Wild type → Wild type EAE mice)

To assess the ability of myeloid *Ikkβ* deletion on transferred EAE, we performed transferred EAE experiment. As respected, EAE symptoms of WT and *LysM-Cre/Ikkβ*^*F/F*^ recipients receiving T cells from *LysM-Cre/Ikkβ*^*F/F*^ donors were significantly lower than those of WT and *LysM-Cre/Ikkβ*^*F/F*^ recipients receiving T cells from WT donors, respectively (Fig. 8a and Table 3). The onsets of EAE in WT (13.6 ± 1.6 days) and *LysM-Cre/Ikkβ*^*F/F*^ recipients (11.6 ± 1.4 days) receiving T cells from *LysM-Cre/Ikkβ*^*F/F*^ donors were delayed than that in WT (7.4 ± 1.6 days) and *LysM-Cre/Ikkβ*^*F/F*^ (9.4 ± 0.2 days) recipients receiving T cells from WT donors, corresponding with decreased maximal clinical score, sum of clinical score, and mortality of EAE (Fig. 8a and Table 3). The overall severity of disease as assessed by cumulative disease index in recipients receiving T cells from *LysM-Cre/Ikkβ*^*F/F*^ donors was significantly lower compared to that in recipients receiving T cells from WT donors (Fig. 8b).

**Table 3:** Myeloid-specific *ikkβ* gene deletion ameliorates transferred EAE

Group	Incidence (%)	Mean day of onset (± SEM)	Maximal clinical score (± SEM)	Sun of clinical score (± SEM)	Mortality (%)
Wild type	5/5	7.4 ± 1.6	4.2 ± 0.7	43.7 ± 10.6	10
→Wild type					
Wild type	5/5	9.4 ± 0.2	3.6 ± 0.2	33.7 ± 2.1	0
→*LysM-Cre/Ikkβ* ^*F/F*^					
*LysM-Cre/Ikkβ* ^*F/F*^	5/5	13.6 ± 1.6*	0.7 ± 0.1**	7.5 ± 2.1**	0
→Wild type					
*LysM-Cre/Ikkβ* ^*F/F*^	5/5	11.6 ± 1.4	1.4 ± 0.2**	12.9 ± 1.8**	0
*→LysM-Cre/Ikkβ* ^*F/F*^					

T cells were isolated from lymph nodes of WT or *LysM-Cre/Ikkβ* ^*F/F*^ donor 15–18 days after induction of active EAE and re-stimulated with MOG_35–55_ peptide. Purified T cells (1 × 10^7^) were transferred i.v. into sub-lethally irradiated WT or *LysM-Cre/Ikkβ* ^*F/F*^ recipient mice. (ANOVA test; *p < 0.05 and **p < 0.01 versus WT → WT group)

## Discussion

In this study, we investigated the in vivo role of IKK/NF-κB-dependent inflammatory myeloid cell activation in EAE using *LysM-Cre/Ikkβ*^*F/F*^ mice. We found that inhibiting inflammatory myeloid cell activation delayed the onset day and alleviated the severity of EAE clinical signs, which was accompanied by reduced demyelination and immune cell infiltration in the spinal cord. The beneficial mechanism of myeloid IKKβ deletion was associated with diminished expansion of Th1 and Th17 cells and increased expression of Treg cells in addition to reduced BBB damage.

In the brain of MS patients or in the EAE animal model, IKK/NF-κB activation is detected in various cell types not only in brain-infiltrating immune and inflammatory cells but also in neurons and oligodendrocytes [41]. The critical role of NF-κB signaling pathways in the development of EAE has long been investigated in in vitro as well as in vivo studies using conventional knockout mice [11, 21]. More recently, the cell-type specific in vivo roles of NF-κB activation in EAE pathology are currently being characterized by different groups using cell type-specific conditional knockout or transgenic mice. For instance, transgenic inactivation of astroglial NF-κB improved the functional and pathological outcomes of EAE by decreasing the astrocyte proinflammatory cytokines and chemokines, indicating the detrimental role of astrocyte NF-κB activation in EAE [3, 4]. Neuronal specific NF-κB ablation through transgenic expression of an IkBα super-repressor (IκBɑ-AA) did not influence neuro-axonal degeneration in EAE, suggesting that neuronal NF-κB signaling is dispensable for EAE [32].

Although research on the cell type-specific function of NF-κB signaling pathways for autoimmune demyelinating disease in CNS cells (neurons and astrocytes) is relatively well-developed, the exact functions of the NF-κB signaling pathways in myeloid cells for MS/EAE remains elusive. In a study, over-expression of the triggering receptor expressed on myeloid cells 2 (TREM2), a NF-κB target gene, in myeloid cells ameliorated clinical symptoms and reduced demyelination as well as axonal damage in the spinal cord of EAE mice, suggesting a neuroprotective role of myeloid NF-κB signaling [56]. In a more recent study, constitutive activation of NF-κB through myeloid specific IkBα deletion resulted in a more severe clinical course of EAE, indicating a detrimental role of myeloid NF-κB activation in EAE development [13]. Our study using *LysM-Cre/Ikkβ*^*F/F*^ mice basically confirmed the previous study by Ellrichmann et al., demonstrating that myeloid NF-κB signalling plays a detrimental role in EAE [13]. Furthermore, in our study, we uncovered the mechanisms underlying these detrimental effects of myeloid NF-κB activation. We found that myeloid NF-κB signaling affects the T cell activation profile during EAE; myeloid IKKβ deletion reduced Th1 and Th17 populations in the spinal cord and lymph nodes, but enhanced the activation of Treg cells in EAE mice. An altered Th cell profile can alleviate demyelination and attenuate the severity of clinical signs of EAE. Therefore, IKKβ/NF-κB-dependent myeloid activation may contribute to EAE pathogenesis by increasing Th1 and Th17 cell responses, while inhibiting Treg cell responses.

The involvement of NF-κB signaling pathways in encephalitogenic T cell activation has been reported in previous studies. In NF-κB/p50-deficient mice, myelin-specific T-cells are unable to differentiate into either Th1- or Th2-type effector cells in vivo [21]. Mice that received mutated NF-κB essential modifier-binding domain (NBD) peptides did not demonstrate a shift in immune responses from a Th1 to a Th2 profile and exhibited reduced encephalitogenicity of myelin-specific T cells [11]. In addition, highly impaired T cell response is observed in splenocyte cultures from T cell specific IKK2 deficient mice [19]. These Th cell profile changes in previous studies were mainly attributed to NF-κB signaling in T cells. In our study, however, we found for the first time to our knowledge that myeloid NF-κB signaling affects the encephalitogenic T cell activation profile in EAE.

In addition, we found that myeloid NF-κB signalling also affects Th17 cell differentiation. Myeloid IKKβ deletion attenuated the mRNA expression of IL-17 and IL-17α, as well as the population of CD4^+^/IL-17^+^ T cells in spinal cords and lymph nodes from EAE mice. NF-κB signalling in T cells has been implicated in Th17 cell activation in previous studies [5, 50]. However, the effect of myeloid NF-κB activation on Th17 cell differentiation has not been reported. Thus far, it is not clear how IKK/NF-κB-mediated myeloid cell activation affect Th1 and Th17 activations. In our study, we observed the Th1/Th17-differentiating effect of myeloid IKK/NF-κB activation both at the spinal cord and the peripheral lymph nodes. Thus, the effects more likely occur during T cell activation in the peripheral lymph node by myeoloid-derived antigen presenting cells (macrophages and myeloid dendritic cells). Indeed, in our study, we found that the expression of various costimulatory molecules and proinflammatory cytokines in the lymph node were down-regulated in the *LysM-Cre/Ikkβ*^*F/F*^ mice, which might be responsible for the reduced Th1 and Th17 cell activation. However, it is also possible that altered chemokine expression in the IKK-deleted spinal cord microglia and macrophage affected the recruitment of Th1 or Th17 cells from the circulation, which was not formally tested in our study. In return, we confirmed that T cells co-cultured with microglia derived from *LysM-Cre/Ikkβ*^*F/F*^ mice displayed lower percentages of Th1 and Th17 cells but higher percentage of Treg cells than those in the control group. Taken together, our findings suggest that myeloid IKKβ can potentiate Th1/Th17 T cell differentiation in the periphery and activation in the spinal lesion of EAE mice, which can exacerbate the clinical severity of EAE.

It is well-known that Treg cells are critical for controlling disease severity in autoimmunity [7, 12, 53, 62]. Treg cells develop in response to TGF-β and express high levels of CTLA-4 and Foxp3 [7, 53, 62]. The critical role of Treg cells in EAE was demonstrated in a study showing aggravated EAE pathogenesis upon Treg cell depletion [24]. We confirmed that myeloid IKKβ deletion significantly up-regulated the number of CD4^+^/CD25^+^/Foxp3^+^ T cells and the mRNA expression of TGF-β and Foxp3 in the spinal cord and lymph nodes after the peak point of EAE clinical signs. Our findings are in line with recent reports showing that artemisinin analogue SM934 [36], dexamethasone [8], and electroacupuncture [38] ameliorated EAE by enhancing the expansion and functions of Treg cells. Of note, the Treg population in peripheral lymph nodes was decreased upon EAE induction, although it increased in the spinal cord. It is likely that the Th1 and Th17 cell proliferation rate in peripheral lymph nodes outweigh the Treg proliferation rate, thus decreasing the relative Treg percentage upon EAE induction. Nevertheless, our results suggest that the EAE-suppressing effects of myeloid IKKβ deletion may be partly due to the increase in the differentiation/activation of Treg cells in the periphery and recruitment/infiltration into the spinal lesion.

Besides the effects on peripheral T cell activation, our data suggest that attenuated BBB compromise may be another mechanism for the EAE-suppressing effects of myeloid IKK/NF-κB signaling. In our study, the EAE-induced BBB leakage measured by Evans blue staining was clearly reduced by myeloid IKKβ deletion, demonstrating that myeloid IKK/NF-κB activation potentiates BBB damage. The effects of myeloid IKKβ on BBB compromise was also confirmed by immunostaining of PECAM-1 and fibronectin proteins, other markers of BBB damage, which were increased in the spinal cords of WT EAE mice in correlation with astrocytic activation. However, the increased immunofluorescence was diminished in *LysM-Cre/Ikkβ*^*F/F*^ mice in agreement with the reduction of astrocytic activation. These data suggest that the suppression of astrocytic activation by myeloid IKKβ deletion may be associated with reduced permeability of the BBB. These findings were similar but distinct from the previous studies in which PECAM-1 and fibronectin were increased in proportion to the level of inflammation in EAE and were observed around capillaries in the CNS of EAE mice [15, 29, 45, 61]. In our study, we found that PECAM-1 and fibronectin are upregulated in activated astrocytes. Although we have not elucidated how myeloid NF-κB activation might have induced astrocyte activation and PECAM-1/fibronectin upregulation, our result suggests putative reciprocal interactions among myeloid cells (specifically, M1/M2 phenotype cells), astrocyte, proinflammatory cytokines/chemokines, cell adhesion molecule, and endothelial cells in the spinal cord. Further studies regarding the molecular interaction of myeloid NF-κB activation and BBB are merited. Since astrocytes are critical in maintaining BBB integrity [51, 60], IKK/NF-κB-mediated myeloid activation might compromise the normal integrity of the BBB by inducing astrocyte activation. Additionally, although pertussis toxin is very effective in permeabilizing an intact BBB [35] and thus allowing myelin-specific T cells to enter the CNS, other mechanisms by which pertussis toxin promotes neuroinflammation in EAE have been suggested [58]. Our results suggest that the enhancement effect of pertussis toxin and inhibitory effect of myeloid IKKβ deletion for permeability of the BBB might be in a mixture in the present study. The BBB of myeloid IKKβ deleted mice might be more resistant to pertussis toxin than WT mice, although the physiologic mechanism remains unclear.

## Conclusions

We found that myeloid cell-specific IKKβ expression plays a pivotal role for demyelination in the EAE model. The inhibition of NF-κB activation by conditional deletion of IKKβ in myeloid cells resulted in alleviated clinical signs of EAE compared with WT mice, corresponding with decreased spinal demyelination and glial activation, decreased infiltration of peripheral immune cells, and reduced expression of proinflammatory mediators. Interestingly, these beneficial effects were associated with the suppression of Th1 and Th17 T cells, the up-regulation of Treg cells, and reduced BBB damage. Our findings imply that therapies targeting IKKβ function in myeloid cells may be effective for the treatment of MS and other demyelinating pathologies of the CNS.

## Abbreviations

BBB, blood–brain barrier; CFA, complete Freund’s adjuvant; CNS, central nervous system; EAE, experimental autoimmune encephalomyelitis; EB, Evans blue; EMEM, Eagle’s minimal essential medium; Iba-1, ionized calcium binding adaptor molecule-1; IKK, IkB kinase; IL, interleukin; iNOS, inducible nitric oxide synthase; LFB, luxol fast blue; LPS, lipopolysaccharide; MOG, myelin oligodendrocyte glycoprotein; MS, multiple sclerosis; NF-κB, nuclear factor kappa B; PECAM-1, platelet endothelial cell adhesion molecule-1; PTX, pertussis toxin; TCR, T cell receptor; TGF-β, transforming growth factor-beta; Th, T-helper; TNF-α, tumor necrosis factor-alpha; Tregs, regulatory T cells; TREM2, triggering receptor expressed on myeloid cells 2; WT, wild-type; ZO-1, zona occluden-1

## Additional files

Additional file 1: PCR primer sequence for PCR analysis. (TIF 93 kb) Additional file 2: Antibodies for immunohistochemical, Western blot, and flow cytometry analyses. (TIF 52 kb) Additional file 3: Additional methods. (DOCX 27 kb) Additional file 4: Myeloid-specific *ikkβ* gene deletion decreases the percentages of CD4^+^, CD4^+^/IFN-γ^+^, and CD4^+^/IL-17^+^ cells but increases the percentages of CD4^+^/CD25^+^/Foxp3^+^ T cells. (A and B) Spinal cords and lymph nodes were dissected from each group (*n* = 5) at day 15–18 post-immunization to investigate the degree of differentiation and recruitment/infiltration of CD4^+^ T cells with flow cytometry. Tissues were dissociated, and cells were incubated with APC anti-mouse CD4, PE anti-mouse CD8α, PE anti-mouse IFN-γ, FITC anti-mouse IL-17A, PE anti-mouse IL-4, PE anti-mouse CD25, and APC anti-mouse Foxp3 antibodies. Far left, CD4^+^ T cell gate (1 × 10^4^ cells in R2 and R15) used to identify CD4^+^/IFN-γ^+^, CD4^+^/IL-17^+^, CD4^+^/IL-4^+^, CD4^+^/Foxp3^+^, and CD4^+^/CD25^+^/Foxp3^+^ T cells. Representative data and mean ± SEM values from 3 independent experiments are shown in spot plot graphs (A, spinal cord; B, lymph nodes). (ANOVA test; **p* < 0.05 and ***p* < 0.01 versus WT EAE mice; #*p* < 0.05 and ##*p* <0.01 versus normal control mice). (TIF 280 kb) Additional file 5: Myeloid-specific *ikkβ* gene deletion reduces disruption of BBB integrity in the spinal cord during peak stage but not onset stage of EAE. After 2 h after Evans blue dye i.v. injection, at onset and peak stages of EAE symptoms, spinal cords were harvested from WT and *LysM-Cre/Ikkβ* ^*F/F*^ EAE mice (*n* = 5 per group), sectioned, and immunofluorescent stained with PECAM-1 (A), Iba-1 (B), and CD68 (C) antibodies. Representative confocal images display extravasated Evans blue dye (a-f in A, B, and C), PECAM-1 (A.g-l), Iba-1 (B.g-i), and CD68 (C.g-i) immunofluorescnce signal, and merged signal (m-r in A, B, and C). Bars = 10 μm. (TIF 389 kb)
