# Supplementary figures and images for: IKKβ-mediated inflammatory myeloid cell activation exacerbates experimental autoimmune encephalomyelitis by potentiating Th1/Th17 cell activation and compromising blood brain barrier
Source: Mol Neurodegener. 2016 Jul 22;11:54. doi: 10.1186/s13024-016-0116-1 (PMC4957872; doi:10.1186/s13024-016-0116-1)

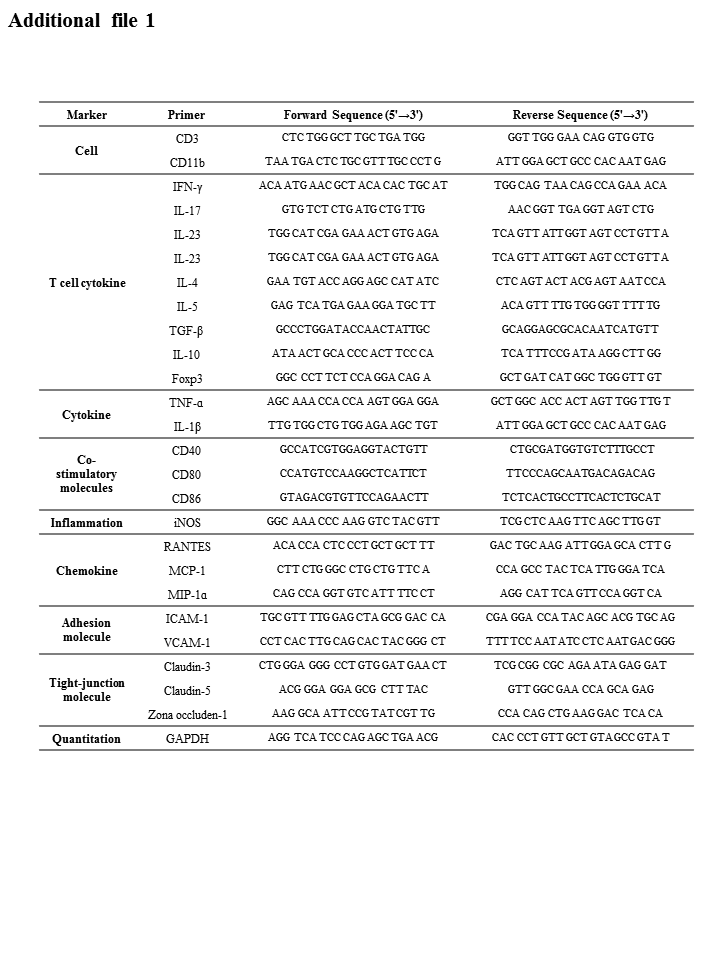

Supplement: Additional file 1: — PCR primer sequence for PCR analysis. (TIF 93 kb) [file 13024_2016_116_MOESM1_ESM.tif]

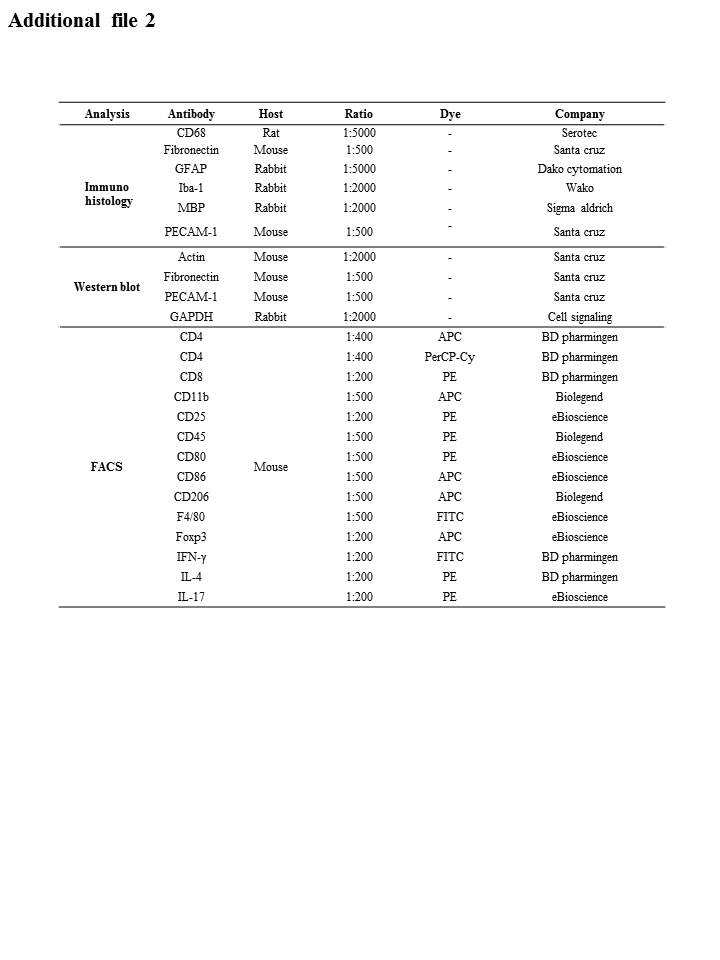

Supplement: Additional file 2: — Antibodies for immunohistochemical, Western blot, and flow cytometry analyses. (TIF 52 kb) [file 13024_2016_116_MOESM2_ESM.tif]

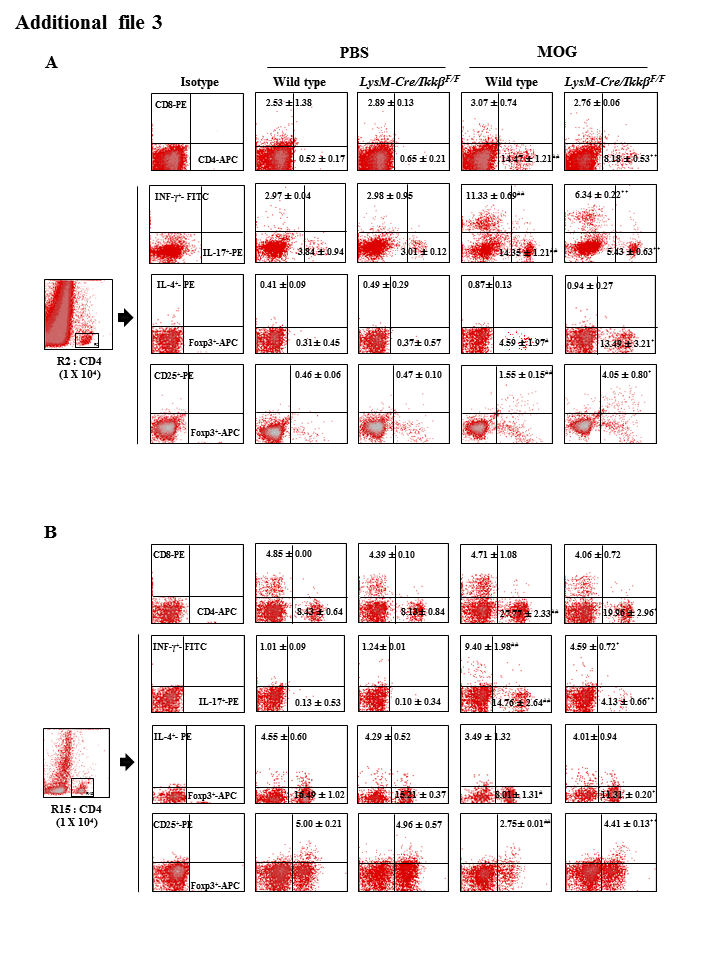

Supplement: Additional file 4: — Myeloid-specific ikkβ gene deletion decreases the percentages of CD4+, CD4+/IFN-γ+, and CD4+/IL-17+ cells but increases the percentages of CD4+/CD25+/Foxp3+ T cells. (A and B) Spinal cords and lymph nodes were dissected from each group (n = 5) at day 15–18 post-immunization to investigate the degree of differentiation and recruitment/infiltration of CD4+ T cells with flow cytometry. Tissues were dissociated, and cells were incubated with APC anti-mouse CD4, PE anti-mouse CD8α, PE anti-mouse IFN-γ, FITC anti-mouse IL-17A, PE anti-mouse IL-4, PE anti-mouse CD25, and APC anti-mouse Foxp3 antibodies. Far left, CD4+ T cell gate (1 × 10^4^ cells in R2 and R15) used to identify CD4+/IFN-γ+, CD4+/IL-17+, CD4+/IL-4+, CD4+/Foxp3+, and CD4+/CD25+/Foxp3+ T cells. Representative data and mean ± SEM values from 3 independent experiments are shown in spot plot graphs (A, spinal cord; B, lymph nodes). (ANOVA test; *p < 0.05 and **p < 0.01 versus WT EAE mice; #p < 0.05 and ##p <0.01 versus normal control mice). (TIF 280 kb) [file 13024_2016_116_MOESM4_ESM.tif]

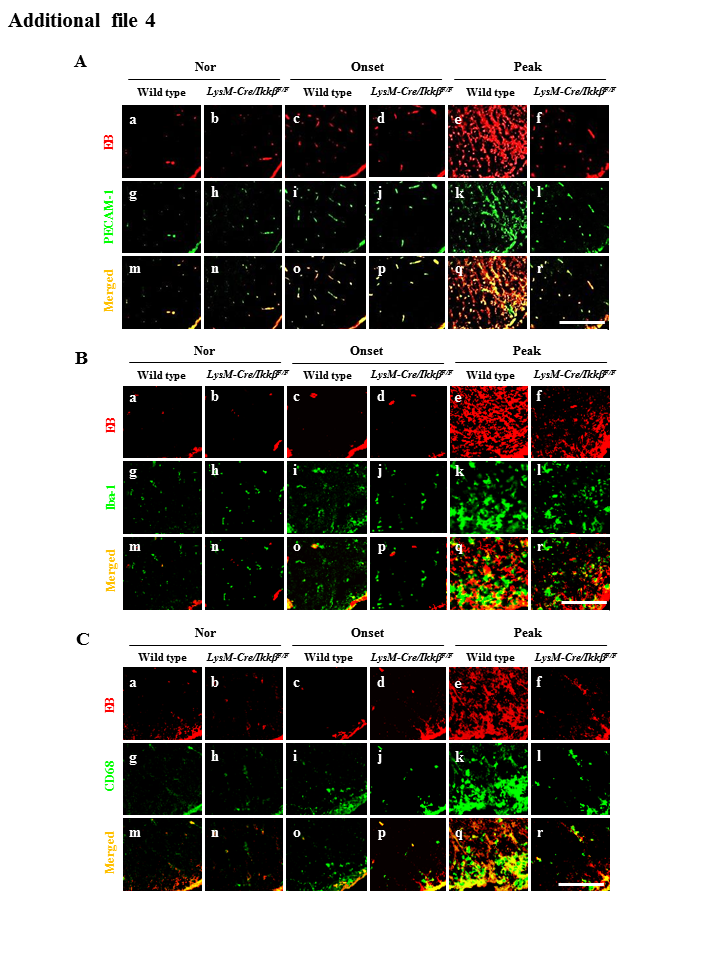

Supplement: Additional file 5: — Myeloid-specific ikkβ gene deletion reduces disruption of BBB integrity in the spinal cord during peak stage but not onset stage of EAE. After 2 h after Evans blue dye i.v. injection, at onset and peak stages of EAE symptoms, spinal cords were harvested from WT and LysM-Cre/Ikkβ F/F EAE mice (n = 5 per group), sectioned, and immunofluorescent stained with PECAM-1 (A), Iba-1 (B), and CD68 (C) antibodies. Representative confocal images display extravasated Evans blue dye (a-f in A, B, and C), PECAM-1 (A.g-l), Iba-1 (B.g-i), and CD68 (C.g-i) immunofluorescnce signal, and merged signal (m-r in A, B, and C). Bars = 10 μm. (TIF 389 kb) [file 13024_2016_116_MOESM5_ESM.tif]
