# Supplementary material for: IKKβ-mediated inflammatory myeloid cell activation exacerbates experimental autoimmune encephalomyelitis by potentiating Th1/Th17 cell activation and compromising blood brain barrier
Source: Mol Neurodegener. 2016 Jul 22;11:54. doi: 10.1186/s13024-016-0116-1 (PMC4957872; doi:10.1186/s13024-016-0116-1)
Supplement: Additional file 3: — Additional methods. (DOCX 27 kb) [file 13024_2016_116_MOESM3_ESM.docx]

**Additional file 3**

**EAE induction and clinical evaluation**

Adult female LysM-Cre/Ikkβ^F/F^ and WT mice were immunized subcutaneously with an emulsion containing 200 µg myelin oligodendrocyte glycoprotein (MOG)_35-55_ peptide (Sigma-Aldrich, St. Louis, MO, USA) and 400 µg of mycobacterium tuberculosis (Difco, Detroit, MI, USA) per ml of Incomplete Freud's Adjuvant (IFA; Difco). Mice (n=3-7 per group) received intraperitoneal injections of 200 ng pertussis toxin (PTX; List Biologic, Campbell, CA, USA) on day 0 of immunization and on day 2 after immunization. Mice in the normal control group were treated with saline alone instead of MOG_35-55_ peptide or PTX. Clinical signs of EAE were evaluated daily and scored using the clinical scoring scale (grade 0, absence of symptoms; grade 1, partial loss of tail tonus; grade 2, paralysis of tail; grade 3, paraparesis; grade 4, paraplegia; grade 5, tetraparesis; grade 6, tetraplegia; grade 7, death). Mice were scored according to their clinical severity with 0.5 points for intermediate clinical findings. Paralyzed mice were assisted with easier access to food and water. Several parameters for EAE were examined to evaluate the severity of EAE including mean clinical score, body weight, incidence, mean day of onset, mean maximal clinical score, sum of clinical score, and mortality (Table 3).

**Quantification of demyelination and cell recruitment/infiltration**

The level of demyelination (by LFB staining) was scored as previously described [1]: 0, no demyelination; 1, little demyelination, only around infiltrates and involving ≤ 25% of the white matter; 2, demyelination involving ≤ 50% of the white matter; and 3, diffuse and widespread demyelination involving ≤ 50% of the white matter. Cell recruitment/infiltration (H&E staining) was evaluated according to the following criteria (Fissolo et al., 2012): 0, no lesion; 1, cellular recruitment/infiltration only in the meninges; 2, very discrete and superficial infiltrates in parenchyma; 3, moderate infiltrate (≤ 25%) in the white matter; 4, severe infiltrates (≤ 50%) in the white matter; 5, more severe infiltrates (≤ 50%) in the white matter.

**Histopathological analysis of spinal cord**

To investigate the effect of myeloid cell type-specific IKK-β-deficiency in normal myelination, adult male mice (10-11 weeks after birth; n=5 per group) were deeply anesthetized with an intraperitoneal overdose of sodium pentobarbital (50 mg/kg, body weight), perfused intracardially with saline, and perfused with a fixative containing 2% paraformaldehyde and 2.5% glutaraldehyde in 0.1 M phosphate buffer (PB, pH 7.4). The lumbar parts of the spinal cords were immediately removed and post-fixed overnight in the same fixative at 4°C. The fixed lumbar segments were rinsed and cut coronally at 2 mm-thickness. The segments were post-fixed in 2% phosphate-buffered osmium tetroxide solution for 2 hours, dehydrated in an ethanol series, passed through propylene oxide, and embedded in epoxy resin. Coronal semithin sections (1.0 μm thick) were placed onto glass slides, dried on a hot plate, and stained with 1% toluidine blue. All sections were captured using a DP70 image analysis system (Olympus, Tokyo, Japan).

For histopathological evidence of EAE, mice (n=8 per group) were anesthetized and then perfused with cold 4% paraformaldehyde in 0.1 m PB on the onset day (7-8 days post-immunization) or after the peak day of neurological impairment (15-18 days post-immunization). The lumbar spinal cords were immediately removed, post-fixed overnight in the same fixative at 4°C, rinsed with PBS, and cryoprotected in 10, 20, and 30% sucrose in PBS serially for 48 hours at 4°C. Coronal sections of 10 μm thickness were prepared on a model CM3050S freezing microtome (Leica, Heidelberger, Nussloch, Germany) and the sections were attached to gelatine-coated slides then stored at −20°C. Histopathological evaluation was performed as described in a recent report [2-4]. Two series of 5 randomly selected sections per spinal cord were processed for evaluation of spinal demyelinatiom using luxol fast blue (LFB) stain and for evaluation of lymphocyte infiltration using hematoxylin and eosin (H&E) stain. Sections were captured using a DP70 image analysis system (Olympus) and analyzed using the NIH Image J program (<http://rsbweb.nih.gov/ij/>).

**Immunohistochemical analysis**

Immunohistochemical analysis was performed as previously described [5]. Briefly, spinal cord sections (10 μm thick) were incubated for 20 minutes with 3% H_2_O_2_ in PBS to remove endogenous peroxidase activity followed by washing in PBS. Sections were then blocked with a solution containing 5% normal goat/or horse serum, 2% bovine serum albumin, 2% fetal bovine serum (FBS), and 0.1% Triton X-100 for 2 hours at room temperature (RT). The sections were incubated overnight at 4˚C with rabbit anti-myelin basic protein (MBP) (1:1,000; Sigma Aldrich), rabbit anti-ionized calcium binding adaptor molecule-1 (Iba-1) (1:2,000; WAKO, Osaka, Japan), and rat anti-CD68 (1:500; Serotec, Oxford, UK). The sections were incubated overnight at 4˚C and washed in PBS. The sections were then incubated with biotinylated rabbit IgG antibody (1:200; Vector Laboratories, Burlingame, CA, USA) for 1 hour at RT. After rinsing, the sections were incubated with avidin-biotinylated horseradish peroxidase (HRP)-complex (1:200; Vector Laboratories) for 1 hour at RT and visualized with 3,3'-diaminobenzidine. The sections were rinsed, dehydrated, and cover-slipped with Permount. For double immunofluorescent staining, sections were incubated overnight at 4°C with a mixture of mouse anti-platelet endothelial cell adhesion molecule [PECAM-1 (CD31); 1: 500; Santa Cruz biotechnology, Santa Cruz, CA, USA), rabbit anti-fibronectin (1: 500; Santa Cruz biotechnology), mouse anti-GFAP (1:500; Dako), and rabbit anti-GFAP (1:500; Millipore, Darmstadt, Germany) antibodies. The sections were incubated for 1 hour at RT with a mixture of Cy3- and FITC-conjugated mouse/rabbit IgG antibodies (1:200; Jackson ImmunoResearch, U.S.A.), and visualized by confocal imaging (LSM 5 PASCAL; Carl Zeiss, Germany).

The regions of interest in each section were captured. Immunopositive cells were quantified as previously detailed [6]. White matter of the lumbar spinal cord was captured from 6 randomly selected sections for each mouse in each group (n = 5). Semiquantitative histological evaluation for Iba-1 antibody (40× magnified microscope field) was conducted and scored blindly by 2 observers using the following scale [6]: resting microglia, mildly activated microglia, and moderately activated microglia.

**T cell culture and proliferation**

Spleen mononuclear cells from WT mice were suspended in culture medium containing Dulbecco's modification of Eagle medium (Gibco, Paisley, UK) supplemented with 1% (v/v) minimum essential medium (Gibco), 2 mM glutamine (Flow Laboratory, Irvine, CA, USA), 50 IU/ml penicillin, 50 mg/ml streptomycin and 10% (v/v) fetal calf serum (both from Gibco). Spleen mononuclear cell suspensions containing 4 × 105 cells in 200 μl culture medium were placed in 96-well round-bottom microtiter plates (Nunc, Copenhagen, Denmark). 10 μl of MOG35–55 peptide (10 μg/ml) or concanavalin A (5 μg/ml; Sigma-Aldrich) was then added to triplicate wells. After 2 and 3 days of incubation, the cells were pulsed for 18 hours with 10 μl aliquots containing 1 μCi of 3H-methylthymidine (specific activity 42 Ci/mmol; Amersham, Arlington Heights, IL, USA). Cells were harvested onto glass fiber filters, and thymidine incorporation was measured. The results were expressed as counts per minute.

**Western blot analysis**

After the peak of neurological impairment, mice (n=5 per group) were anesthetized, and the lumbar spinal cords were removed with lysis buffer (50 mm Tris-Cl, pH 7.5, 150 mm NaCl, 1% Triton X-100, 10% glycerol, and protease inhibitor mixture). Western blot analysis was performed as previously described [2-4]. Briefly, proteins were transferred to polyvinylidene fluoride membranes and the membranes were probed overnight with primary antibodies (Table 1) at 4°C, followed by incubation with HRP-conjugated secondary antibody at RT for 1 hour prior to enhanced chemiluminescence (Amersham Pharmacia Biotechnology, Piscataway, NJ, USA) treatment and exposure to X-ray film. For normalization of the antibody signal, the membranes were stripped and reprobed with antibodies for actin (1:2,000; Cell Signaling Technology) or glyceraldehyde 3-phosphate dehydrogenase (GAPDH) (1:1000; Cell Signaling Technology). After Western blotting was performed several times, the density of each band was converted to numerical values using the Photoshop CS2 program (Adobe, San Jose, CA, USA), with the background values subtracted from an area of film immediately adjacent to the stained band. Data are expressed as the ratio of MBP, PECAM-1, and fibronectin against actin or GAPDH for each sample.

**Normalization of the antibody signal by Western blot analysis**

For normalization of the antibody signal, the membranes were stripped and reprobed with antibodies for actin (1:2,000; Cell Signaling Technology) or glyceraldehyde 3-phosphate dehydrogenase (GAPDH) (1:1000; Cell Signaling Technology). After Western blotting was performed three times, the density of each band was converted to numerical values using the Photoshop CS2 program (Adobe, San Jose, CA, USA), with the background values subtracted from an area of film immediately adjacent to the stained band. Data are expressed as the ratio of proteins against actin or GAPDH for each sample.

**References**

**1.** Fissolo N, Costa C, Nurtdinov RN, Bustamante MF, Llombart V, Mansilla MJ, Espejo C, Montalban X, Comabella M. [Treatment with MOG-DNA vaccines induces CD4+CD25+FoxP3+ regulatory T cells and up-regulates genes with neuroprotective functions in experimental autoimmune encephalomyelitis.](http://www.ncbi.nlm.nih.gov/pubmed/22727044) J Neuroinflammation. 2012 Jun 22;9:139.

**2.** Jang M, Lee MJ, Cho IH. Ethyl pyruvate ameliorates 3-nitropropionic acid-induced striatal toxicity through anti-neuronal cell death and anti-inflammatory mechanisms. *Brain, behavior, and immunity*. 2014; **38:** 151-165

**3.** Lee MJ, Jang M, Choi J, Chang BS, Kim DY, Kim SH, Kwak YS, Oh S, Lee JH, Chang BJ, Nah SY, Cho IH. Korean red ginseng and ginsenoside-Rb1/-Rg1 alleviates experimental autoimmune encephalomyelitis by suppressing Th1 and Th17 T cells and upregulating regulatory T cells. *Molecular neurobiology.* 2016 Apr;53(3):1977-2002.

**4.** Lee MJ, Jang M, Choi J, Lee G, Min HJ, Chung WS, Kim JI, Jee Y, Chae Y, Kim SH, Lee SJ, Cho IH. Bee Venom Acupuncture Alleviates Experimental Autoimmune Encephalomyelitis by Upregulating Regulatory T Cells and Suppressing Th1 and Th17 Responses. *Molecular neurobiology.* 2016 Apr;53(3):1419-45.

**5.** Cho IH, Hong J, Suh EC, Kim JH, Lee H, Lee JE, Lee S, Kim CH, Kim DW, Jo EK, Lee KE, Karin M, Lee SJ. Role of microglial IKKbeta in kainic acid-induced hippocampal neuronal cell death. *Brain*. 2008; **131:** 3019-3033

**6.** VanGuilder HD, Bixler GV, Brucklacher RM, Farley JA, Yan H, Warrington JP et al. Concurrent hippocampal induction of MHC II pathway components and glial activation with advanced aging is not correlated with cognitive impairment. Journal of neuroinflammation 2011; 8: 138.
